# Human gnathostomiasis – A systematic review and analysis of the literature

**DOI:** 10.1371/journal.pntd.0014546

**Published:** 2026-07-31

**Authors:** Simon Frey, Esther Kuenzli, Lars Henning, Francisco Bravo, Yukifumi Nawa, Andreas Neumayr

**Affiliations:** 1 Swiss Tropical and Public Health Institute, Basel, Switzerland; 2 University of Basel, Basel, Switzerland; 3 College of Medicine and Dentistry, James Cook University, Queensland, Australia; 4 Instituto de Medicina Tropical Alexander von Humboldt, Universidad Peruana Cayetano Heredia, Lima, Peru; 5 Khon Kaen University, Khon Kaen, Thailand; Consejo Nacional de Investigaciones Cientificas y Tecnicas, Fundación Mundo Sano, ARGENTINA

## Abstract

Human gnathostomiasis is a foodborne zoonotic nematode infection caused by the larval stage of *Gnathostoma* species. Although historically reported predominantly from Asia and America, cases are increasingly identified in previously non-endemic regions. The heterogeneous clinical presentation and diagnostic challenges likely contribute to underrecognition of the global disease burden. Current evidence is largely derived from case reports and case series, with a notable lack of prospective studies. To better delineate the epidemiological distribution and clinical characteristics of human gnathostomiasis, we systematically reviewed and analyzed the available literature. Most cases are reported from Asia and the Americas, with Thailand, Japan, and Mexico accounting for the highest numbers. Clinical manifestations are predominantly larva migrans syndromes, including cutaneous involvement (83.6% of cases), followed by neurological (7.7%), ocular (3.5%), and visceral (2.3%) presentations. Long-term sequelae are uncommon overall but occur frequently in ocular (51.8%) and neurological (32.2%) disease, the latter also being associated with a substantial case-fatality rate (33.9%). Diagnosis is based on exposure risk, most commonly consumption of raw fish (70.7%), as well as laboratory findings such as eosinophilia, positive serology, and, when feasible, histopathological confirmation. Treatment primarily relies on anthelmintic treatment with albendazole and/or ivermectin and, if feasible, surgical removal of larvae. Treatment success is highest with albendazole plus ivermectin combination therapy (86.7%) compared to monotherapy with albendazole (73.5%) or ivermectin (56.7%).

## Author summary

Human gnathostomiasis is a tropical parasitic worm infection that humans usually acquire by eating raw or undercooked fish. While most infections are reported in Asia (especially Thailand and Japan) and America (especially Mexico), there are increasing reports of cases from countries where the disease was previously unknown. The symptoms are caused by the larval stages of *Gnathostoma* worms, which migrate through the human body after ingestion. Most commonly, the larvae migrate under the skin, but in general, almost any part of the body or organ can be affected. Treatment is with antiparasitic drugs, as surgical removal of the larvae is only possible if they migrate very superficially. Because symptoms can vary greatly, depending on the part of the body or organ affected, the disease is difficult to diagnose, and many cases may go undetected. Most of the available information about the disease comes from individual case reports and small case series, rather than large-scale studies. To better understand where the disease occurs and how it presents, is diagnosed, and is treated, we systematically reviewed and analyzed the published literature and combined the available data to obtain a clearer overall picture of this disease.

## Introduction

Human gnathostomiasis is a foodborne parasitic zoonosis caused by tissue-invasive spirurid nematode larvae of various *Gnathostoma* species. The parasite was first described in 1836 by Sir Richard Owen, who recovered the adult parasite from a gastric wall mass of a tiger which died at the London Zoological Gardens [1]. In 1889, the first human case was published, reporting the recovery of the parasite’s larval stage from a breast abscess of a female Thai patient [2]. The parasite’s complete life cycle was finally described by Prommas and Daengsvang in 1937 [3].

### Life cycle

**Fig 1:**
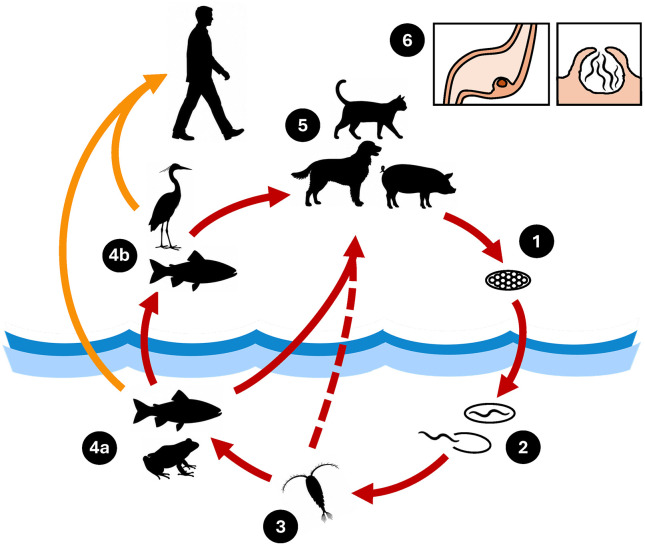
Life cycle of *Gnathostoma* spp. (1) Unembryonated eggs are shed with the definitive host’s feces into fresh water; (2) Eggs embryonate and early first stage larva (EL1) develops and hatches; (3) Early first stage larva (EL1) infects first intermediate host and develops into early third stage larva (EL3); (4a) Early third stage larva develops in the second intermediate host into advanced third stage larva (AL3); (5) The definitive host is infected by consumption of AL3 harboring second intermediate (4a) or paratenic hosts (4b); (6) Adult parasites lodge and mate in tumorous cavities they form (depending on the *Gnathostoma* species) in the stomach or esophagus wall. [Fig 1 includes graphical silhouettes generated using OpenAI’s ChatGPT image generation system (GPT-5.5; available at: https://chatgpt.com/. Accessed July 13th, 2026)].

**Fig 2:**
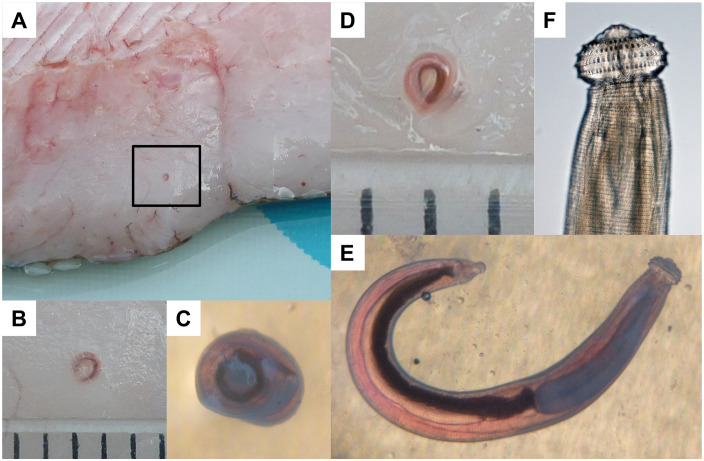
*Gnathostoma binucleatum* third stage larva. **(A-D)** Advanced third-stage larva (AL3) of *Gnathostoma binucleatum* encysted in the muscle tissue of a second intermediate host freshwater fish (the bottom scale in **(B)** and **(D)** depicts millimeters); **(E)** Advanced third-stage larva (AL3) of *Gnathostoma binucleatum* (length: ~ 1.5–2mm); **(F)** Characteristic cephalic region of the advanced third-stage larva (AL3) of *Gnathostoma binucleatum* covered by four transverse rows of cuticular spines and the adjacent body covered with transverse rows of flat spines [Images: Neumayr A].

The life cycle of the *Gnathostoma* spp. typically requires two intermediate hosts and a definitive host (Fig 1). Definitive hosts are wild and domestic carnivorous or omnivorous mammals in which male and female adult worms (size: female: ~ 2–10 cm; male: ~ 1–6 cm) live in tumorous cavities they form (depending on the *Gnathostoma* species) in the hosts’ stomach, esophagus wall or urinary system. Adults mate and produce unembryonated eggs, which are shed in the host’s feces. Eggs become embryonated in fresh water and early first-stage larvae (EL1) hatch. Freshwater copepods (*Cyclops* spp.), which serve as first intermediate hosts, ingest the free-swimming EL1, and the larvae molt twice to become early third-stage larvae (EL3). Following ingestion of the copepod by a suitable second intermediate host (i.e., fish or amphibian), EL3 migrate into the tissues of the host where they develop into advanced third stage larvae (AL3) and encyst. The definitive host is infected by consumption of AL3 harboring second intermediate or paratenic hosts [4,5]. Fig 1 shows the parasite’s life cycle, Table 1 lists the typical intermediate and definitive host species of the human pathogenic *Gnathostoma* species, and Fig 2 shows some photographic examples of AL3 larvae.

**Table 1:** Life cycle characteristics and epidemiology of human pathogenic *Gnathostoma* spp.

*Gnathostoma* spp.	Second intermediate host species^1^	Definitive host species	Anatomic location of the adult parasite(s) in the definitive host	Geographic distribution of reported human cases
*G. spinigerum*	fish, reptiles, amphibians, birds	cats (and other wild felines), dogs, civet, mink, opossum, otter, raccoon	stomach	Thailand, Japan, China, India, Sri Lanka, Myanmar, Cambodia, Laos, Vietnam, Malaysia, Indonesia, the Philippines, Bangladesh, Korea, Nepal, Australia, New Zealand, Zambia, Botswana, South Africa, Madagascar
*G. binucleatum*	fish, turtles	cats, dogs, ocelot	stomach	Mexico, Ecuador, Peru, Guatemala
*G. doloresi*	fish, reptiles, amphibians	pig, wild boar	stomach	Japan^2^, Thailand, Vietnam, the Philippines, China
*G. hispidum*	fish, birds, amphibians, mammals	pig, wild boar	stomach	Japan^2^, Thailand, Vietnam, Cambodia, the Philippines, China, Spain
*G. malaysiae*	*unknown*	rodents	stomach	Myanmar^3^
*G. nipponicum*	fish, reptiles, amphibians	weasel	esophagus	Japan^2^, China, Korea

^1^The list of intermediate/paratenic and definitive host species is not exhaustive and limited to the most important examples. Note that in Thailand alone, 48 species of vertebrates (including 20 species of freshwater fish) have been identified as intermediate and/or paratenic hosts and in Mexico, 25 species of fish and seven species of fish-eating birds have been identified as intermediate/paratenic hosts [8].

^2^Note: despite the widespread distribution of *G. doloresi, G. hispidum*, and *G. nipponicum* across Asia, human infections are reported almost exclusively from Japan.

^3^Only one *G. malaysiae* infection in humans is described to date, a Japanese traveler possibly infected in Myanmar

To date, 14 valid species of the genus *Gnathostoma* are described [6,7], six are known to be human pathogenic (Table 1).

Humans are accidental dead-end hosts, classically acquiring the infection by the consumption of raw or undercooked meat of second intermediate or paratenic hosts [5,9]. In addition, drinking water containing infected copepods and handling meat from infected secondary intermediate hosts with bare hands are also considered possible routes of human infection [10,11]. Furthermore, the description of an infected 3-day-old infant suggests the possibility of intrauterine/perinatal transmission [12].

### Epidemiology

Human gnathostomiasis is endemic mainly in Asia and the Americas. In Asia, most cases of human gnathostomiasis have been reported from Thailand (cumulatively more than 9,000 cases) and Japan (cumulatively more than 4,000 cases), but cases are documented across most of Asia (from India and Sri Lanka to China, the Philippines, and Indonesia) [6]. In the 1970s, gnathostomiasis was discovered to also be endemic in certain regions of Mexico [13] with more than 10,000 human cases reported since [6]. In 1979, human cases of gnathostomiasis emerged in Ecuador (with more than 2,000 cases reported since [6]) and following the first reported case series in 2001 [14], cases continue to also be reported from Peru [11,14–17]. Sporadically, cases are reported from other Latin American countries [18] and North America [19,20]. In Australia, the first suspected case of autochthonously acquired gnathostomiasis was reported in 1970 [21], followed by sporadic case reports since [22]. Although no cases of gnathostomiasis have been reported among the local African population to date, individual case reports of infections diagnosed in travelers returning from Africa indicate that the parasite is also present in sub-Saharan Africa [23–28]. In Europe, locally acquired gnathostomiasis is limited to two cases reported from Spain [29]. The high incidence of infection in certain regions is primarily attributable to specific regional dietary habits and typical local raw dishes, such as, e.g., *koi pla* in Thailand, *koi ga* in Vietnam, *sashimi* and *sushi* in Japan, and *ceviche* in Latin America.

### Clinical presentation

**Fig 3:**
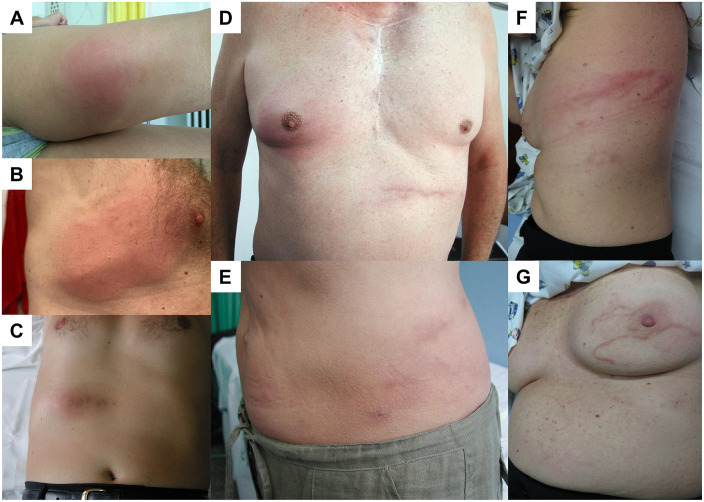
The different types of skin lesions observed in human gnathostomiasis. Examples of cutanous larva migrans (CLM) syndrome/ creeping eruptions in patients with *Gnathostoma binucleatum* infection: Depending on the depth of the parasite’s tissue migration, the morphology of the lesions ranges from erythematous swellings **(A–D)** to superficial serpiginous migration tracks **(D–G)**. [Images: Neumayr A **(A)**, Bravo F **(B–G)**].

In endemic areas, infections in humans are often so common that the disease is known by specific local names among the population: e.g., *Tua-chid* (Thailand), *Choko-fushu* (Japan), *Rangoon tumor* (Myanmar), *Yangtze River edema* or *Shanghai rheumatism* (China), *Woodbury bug* (Australia). The symptoms observed in human gnathostomiasis reflect the erratic tissue migration of AL3 in the accidental human host. Although AL3 larvae may mature to young adults in the human host, patency is not reached [30]. Since the life span of larvae is limited, the natural death of the parasite limits the duration of the infection. However, untreated human infection may last for several years, with symptoms lasting over 10 years [4] and up to 17 years [31] being reported. The larvae migrate through the host tissue without specific organ tropism at a speed of up to 3 cm per hour [6], with the migration path being reflected in the symptoms caused. In general, human gnathostomiasis primarily presents as cutaneous larva migrans (CLM) and visceral larva migrans (VLM) syndrome. CLM is characterized by subcutaneous migratory erythema or swellings in cases of deep tissue migration and by serpiginous creeping eruptions in cases of more superficial tissue migration (Fig 3). *Gnathostoma spinigerum* and *G. binucleatum* are reported to predominately cause migratory erythema and swellings with a predilection for the extremities and the face/head and symptoms lasting up to several years, while *G. hispidum*, *G. nipponicum* and *G. doloresi* predominantly present with serpiginous creeping eruptions with a predilection for the body trunk and symptoms being limited to a few months [6].

VLM may affect any organ, including the central nervous system (CNS). Although CNS involvement is relatively rare, neural larva migrans (NLM; neurognathostomiasis) and ocular larva migrans (OLM; ocular gnathostomiasis) are feared due to the potentially serious consequences [32,33]. Interestingly, neurognathostomiasis is almost exclusively reported from Thailand and rarely from other countries, while ocular gnathostomiasis is almost equally reported in Thailand, Japan, India, Myanmar and Mexico [6].

### Diagnostics

**Fig 4:**
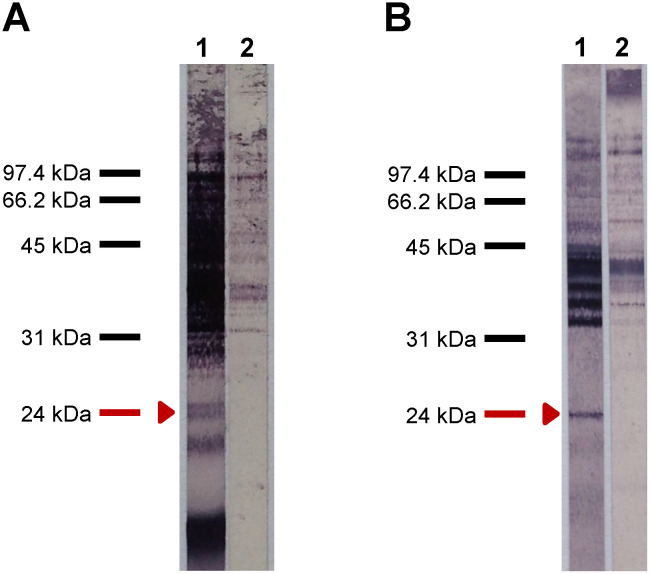
Westernblot diagnostic of human gnathostomiasis infection. **(A)** Western blot based on *G. spinigerum* third stage larva (AL3) crude antigen preparation: Lane 1: serum of a Laotian patient with *G. spinigerum* infection; Lane 2: negative control; **(B)** Western blot based on *G. binucleatum* third stage larva (AL3) crude antigen preparation: Lane 1: serum of a Peruvian patient with *G. binucleatum* infection, Lane 2: negative control.

Diagnosing gnathostomiasis relies on plausible exposure history (epidemiology and source of infection), compatible clinical symptoms (primarily the clinical picture of CLM), and specific laboratory testing. Regards to the latter, a definitive diagnosis, relying on the identification of the parasite by microscopy in biopsy specimen is, besides the occasional spontaneous percutaneous emergence of the larva, only rarely possible. The presence of eosinophilia, in blood or tissue biopsies, may support the diagnosis of gnathostomiasis but is neither reliably sensitive nor specific. Histopathologic findings may demonstrate superficial and/or deep perivascular eosinophilic infiltrates, with eosinophils comprising up to 95% of inflammatory cells in later lesion stages [34]. In the majority of cases, the laboratory evidence is limited to indirect supportive serological tests, detecting *Gnathostoma*-specific antibodies in the patient’s serum [5]. Before modern serological assays became available in the 1970s, skin testing, based on the intracutaneous injection of purified AL3 antigen provoking a delayed-type (cell-mediated) hypersensitivity reaction in sensitized persons, was used in endemic areas [4,35]. With the development of immunodiagnostics, skin testing was replaced by ELISA and later by immunoblot (Western blot), which to date is the most widely used test. The immunoblot detects IgG antibodies in the patient’s serum, specifically targeting a 24 kDa polypeptide of *G. spinigerum-* and G. *binucleatum*-AL3 crude antigen extract (Fig 4) [36–40]. Although well established in endemic countries, the limited availability of serological tests in non-endemic countries often restricts the diagnostic options. Finally, although equally restricted with regards to availability, polymerase chain reaction (PCR) and sequencing techniques may also be helpful to establish the diagnosis and determine the causative *Gnathostoma* species in biopsy material [41,42].

### Treatment and outcome

Besides the few cases where manual removal of very superficially migrating *Gnathostoma* larva allows for manual/surgical removal, the main treatment of human gnathostomiasis is medically, either with albendazole [43–46], with ivermectin, or with a combination of both drugs. Although failures of medical treatment are not infrequently observed [47] and frequently several rounds of treatment may be necessary to achieve cure, the prognosis of *Gnathostoma*-related CLM and VLM is generally good. The prognosis of *Gnathostoma*-related NLM and OLM is less favorable, as permanent sequelae and, in severe cases, even death can result.

With this systematic review and analysis of the literature on human gnathostomiasis, we aim to provide clinicians with a comprehensive summary of the available data, focusing on the clinically relevant core aspects of the disease.

## Methods

This systematic review was registered prospectively in PROSPERO (CRD42024613813). A systematic literature search was performed of the databases CINAHL, Cochrane, EMBASE Elsevier, Medline, PubMed, Scopus and Web of Science on 14/Nov/2024 with the following search term ((Gnathostoma [Mesh] OR Gnathostomiasis [Mesh])) OR (Gnathostom* [tiab] OR “G. spinigerum” [tiab] OR “*G. hispidum*” [tiab] OR “*G. doloresi*” [tiab] OR “*G. nipponicum*” [tiab] OR “*G. malaysiae*” [tiab] OR “*G. binucleatum*” [tiab]) NOT (“Animals” [Mesh] NOT “Humans” [Mesh]), with adaptions according to the corresponding databases. A detailed description of the search strategy can be found in S1 Text. After duplicate removal through EndNote (version 21, 2013, Philadelphia, PA, Clarivate), Covidence (https://www.covidence.org/) and manually, all publications were pre-screened by title and abstract. All studies not concerning human gnathostomiasis or not matching the inclusion criteria (clinical cases with data on epidemiology, diagnostics, treatment or outcome) were excluded. All remaining studies were full text screened according to the inclusion criteria in the systematic review protocol and studies not matching were excluded. Non-retrievable studies, neither available through the corresponding journal, nor through library services nor direct contacting of the author were labelled “non retrievable” and excluded. In the full text review stage, the reference lists of the reviewed studies were checked for potentially eligible studies not detected earlier (*snowball-search* approach). If a review contained clinical data of cases from non-retrievable or foreign language sources, the data were included with the remark “as referenced in”. A second search was conducted on 20/Aug/2025 with the identical search string to detect and include the most recent publications. From the included studies, the following data were extracted: Author, publication title, publication date, journal, country of study, type of study, study period, language, number of gnathostomiasis cases, patients age, sex, country of acquisition, minimum incubation period, country of patients origin, autochthonous or imported case, expositional risk factors, preexisting medical conditions, immunosuppression, pregnancy, symptoms, duration of hospitalization, number of larvae, clinical syndrome (CLM, VLM, NLM, OLM), affected body part, laboratory values, diagnostic testing (serology, skin test, PCR), location of laboratory, histology, *Gnathostoma* species, imaging findings, treatment, treatment induced eruption, larva removal, used anthelmintic(s), dosage, duration, retreatment, side effects, relapse, number of relapses, outcome, complications, sequelae. Uncertainties during the review process were resolved by consulting a second reviewer. The used data extraction sheet can be found in S1 Table.

## Results

**Fig 5:**
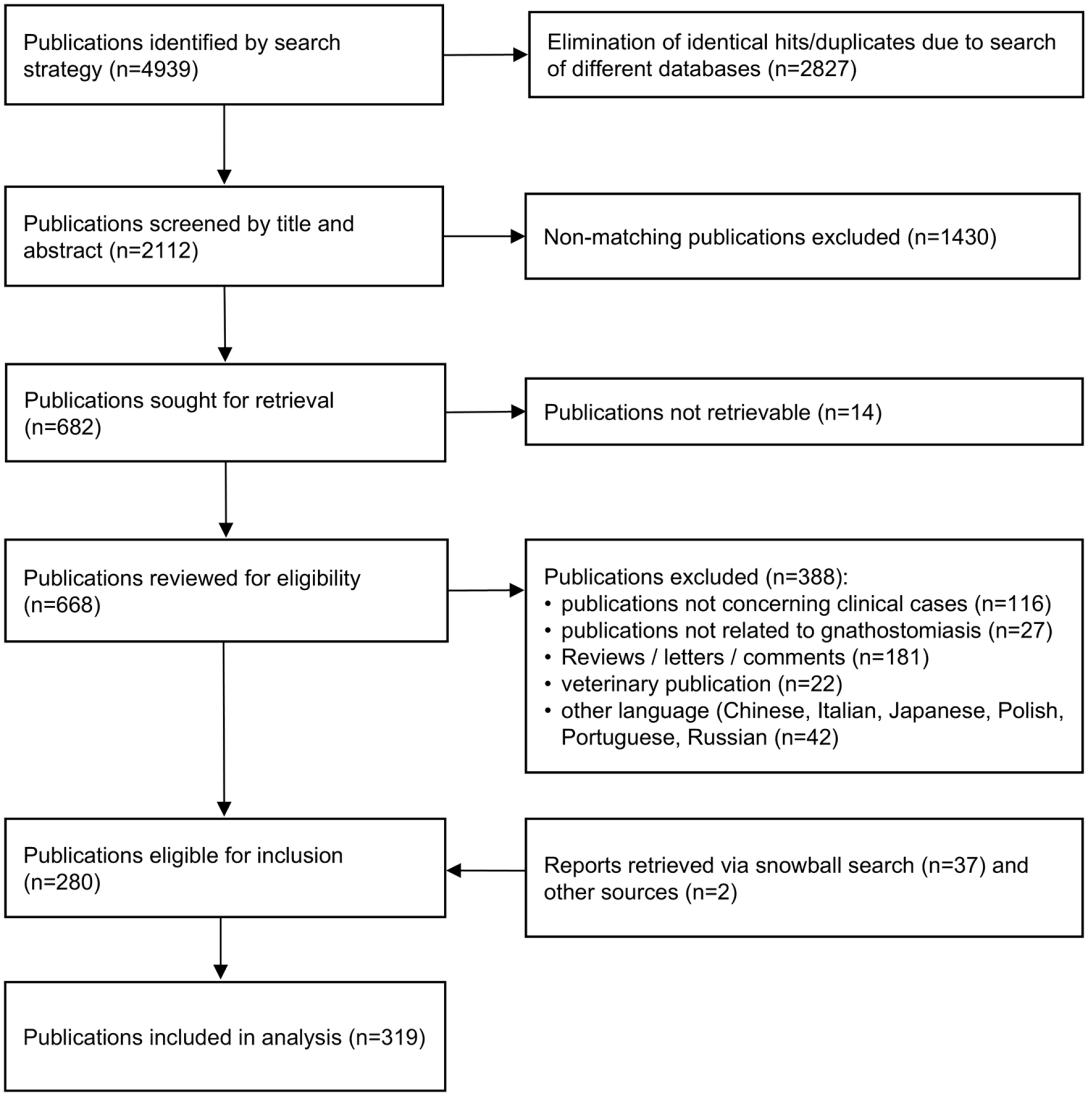
Flow diagram of search and selection of eligible publications.

Our search identified 4,939 publications, of which 319 proved eligible for inclusion in the review (Fig 5). The reference lists of the included publications and the PRISMA checklist for systematic reviews are available in S2 Text.

In the 319 reviewed papers, a total of 2,433 gnathostomiasis cases were reported. Considering the level of diagnostic certainty, we defined respective categories according to which these cases can be divided into 1,221 “possible cases” (Def.: compatible clinical picture), 1,016 “probable cases” (Def.: compatible clinical picture + positive serology), and 196 “confirmed cases” (Def.: detection of parasite by microscopy or by polymerase chain reaction (PCR) in biopsy).

In 16 patients, multiple lesions of migrating larvae were present at the same time. The reported number of lesions/larvae in these cases were: N_lesions/larvae_ (N_patients_): 2 (10), 3 (1), 4 (2), 5 (2), 12 (1).

## Discussion

**Fig 6:**
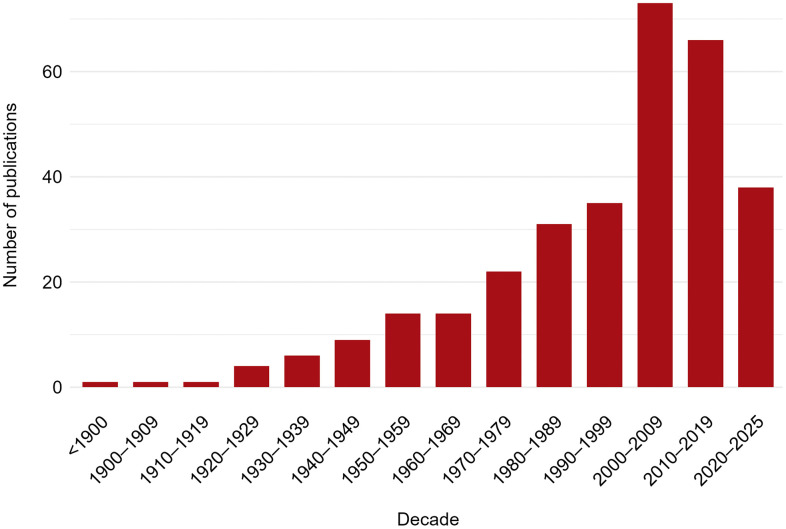
Number of publications reporting human gnathostomiasis cases from 1890–2025 (N = 319).

**Fig 7:**
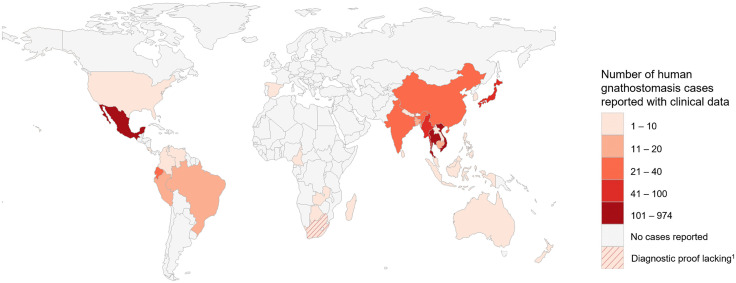
Geographic distribution of the analyzed cases with available individual clinical and epidemiological data (N = 2257). ^1^ From South Africa a single suspected case of gnathostomiasis has been reported as personal communication [48]. [Map generated in R using the choroplethr package with basemap shapefiles obtained from Natural Earth Data (Link to source: https://www.naturalearthdata.com/downloads/; Link to terms of use: http://www.naturalearthdata.com/about/terms-of-use/)].

Since the discovery of the parasite in 1889, the number of publications on human gnathostomiasis has increased slowly but steadily, peaking between 2000 and 2010 (Fig 6), the period during which most clinical treatment studies were also conducted. [44,50,51,53]. Most cases of gnathostomiasis in humans continue to be reported from the traditional high endemic regions in Asia and Latin America. Over the years, the sporadic reports of diagnosed infections in returning travelers [23–25,28] as well as autochthonous infections in regions previously considered non-endemic [21,22,26,29] have expanded the known geographic range of the parasite (Fig 7).

The level of diagnostic certainty of the reviewed cases of gnathostomiasis varies considerably. Among the reviewed 2433 clinical cases only 196 were “confirmed”, while 1016 were “probable” and 1221 “possible” cases. Since confirmation of gnathostomiasis in humans is limited to the few cases in which the parasite can be detected and diagnosed using direct methods (microscopy, PCR), it is not surprising that most cases remain unconfirmed, and the diagnosis based on the clinical picture and/or the indirect method of serology. The “probable” category is also at risk to misclassification bias, particularly in regions where cross-reactivity with other parasites is possible, which can potentially limit the comparability across studies. Furthermore, different assay types, antigen source and inconsistent availability of serology across regions further limits the accuracy of the classification. We assessed the possibility of a stratified subgroup analysis between “confirmed,” “probable,” and “possible” cases; however, the number of “confirmed” cases was largely insufficient to perform such an analysis. Consequently, it is possible that some of the “probable” or “possible” cases in the original publications were incorrectly classified as gnathostomiasis, which confounds the validity of our analyses. Although this limitation has to be acknowledged, we nevertheless consider our data set to be the best available.

**Fig 8:**
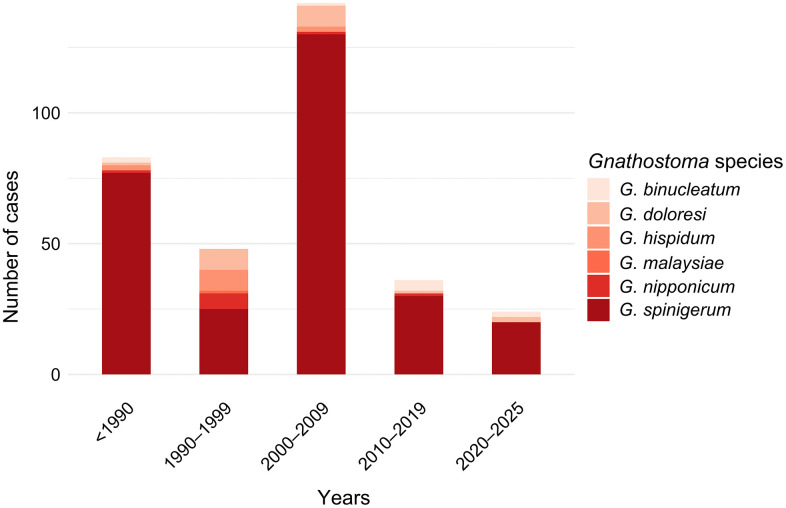
Number of reported cases with available individual clinical data by species over time (N = 333).

The vast majority of human gnathostomiasis is caused by *G. spinigerum* (Fig 8) and primarily originate from Asia with some sporadic cases also being reported from Australasia and Africa. Although early morphological studies of larvae obtained by biopsy from patients in Mexico and Ecuador described the larvae to be similar to those of *G. spinigerum* [54–57] later molecular genetic analysis revealed that human gnathostomiasis in Mexico and Ecuador is exclusively caused by the morphologically very similar species *G. binucleatum* [58,59]. To date, *G. binucleatum* is the only known zoonotic *Gnathostoma* species prevalent in the Americas, and the second most frequent *Gnathostoma* species causing human gnathostomiasis [6]. In this regard, it should be noted that the latter fact is well reflected in Fig 7 (with the high number of cases reported from Mexico) but not in Fig 8. Fig 8 is based on ‘reported cases with available individual clinical data’ and as such data is missing for most cases reported from Mexico, these are not included in the figure. All other human pathogenic G*nathostoma* spp. (Table 1) are considerably less frequent (Table 5) and, besides two reported human *G. hispidum* cases from Spain [29], exclusively reported from Asia.

As expected, raw fish was mostly cited as the suspected source of infection (Table 2), although the consumption of other intermediate and paratenic host species, as well as contaminated water (containing infected copepods), were also cited as suspected sources of infection.

**Table 2:** Possible sources of infection reported in the investigated cases of gnathostomiasis in humans, including mention of various possible sources of infection (N = 378).

Possible source of infection	N	%
Raw fish	267	70.7
Raw meat (*not specified*)	17	4.5
Water containing infected copepods	15	4.0
Raw amphibia	13	3.5
Raw loach	10	2.6
Raw pork	10	2.6
Raw snake	10	2.6
Raw poultry	8	2.1
Raw beef	6	1.6
Raw shrimp	6	1.6
Raw snail	5	1.3
Raw crab	2	0.5
Raw eel	2	0.5
Raw food (*not specified*)	2	0.5
Raw prawn	2	0.5
Raw boar	1	0.3
Raw lobster	1	0.3
Raw oysters	1	0.3

The primary clinical manifestation of human gnathostomiasis is CLM caused by a single migrating larva. The concomitant presence of more than one migrating larva in a patient is overall rare and was reported in only 0.7% (16/2421) of the cases (N_lesions/larvae_ [N_patients_]: 2 [10], 3 [1], 4 [2], 5 [2], 12 [1]).

Most CLM lesions manifest as subcutaneous swelling (73.4%) or serpiginous dermal tracks (9.7%), reflecting deeper or more superficial larval migration, respectively (Table 3).

**Table 3:** Reported morphology and associated symptoms of gnathostomal CLM lesions (N_symptoms_ = 1684).

Morphology and associated symptoms of gnathostomal CLM lesion	Reported frequency N (%)
Subcutaneous swelling	1236 (73.4)
Serpiginous dermal track	163 (9.7)
Localized itching	135 (8.0)
Localized pain	88 (5.2)
Plaque	24 (1.4)
Nodule	21 (1.2)
Localized urticarial rash	11 (0.7)
Papule	4 (0.2)
Localized hemorrhage	1 (0.1)
Localized rash	1 (0.1)

CLM, cutaneous larva migrans.

**Fig 9:**
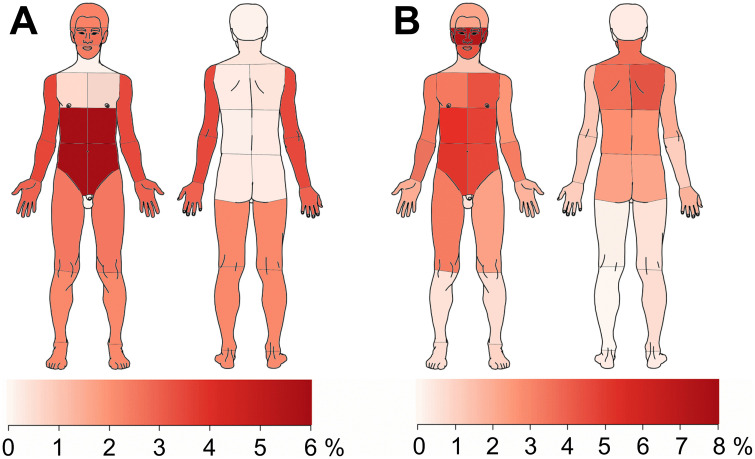
Anatomical localization of gnathostomal CLM lesions (N = 7890) in the reviewed cases with available individual data (N = 1276). **(A)** CLM lesions which descriptions allowed only for a rough assignment to the main anatomical regions of the body (N = 7325); **(B)** CLM lesions which descriptions allowed for a detailed assignment to anatomical subregions of the body (N = 565); [Body template adapted from an open source clipart (Link to source: https://openclipart.org/detail/314881/human-male-and-female-body-line-art)].

**Fig 10:**
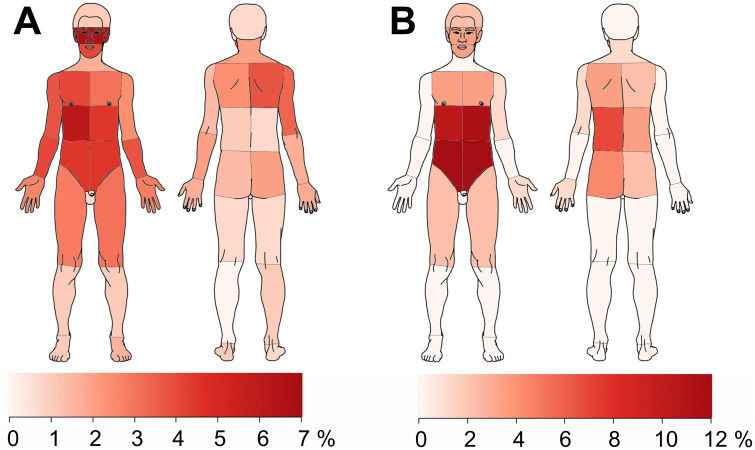
Anatomical localization of gnathostomal CLM lesions: CLM lesions due to *Gnathostoma spinigerum* and *G. binucle*atum vs. CLM lesions due to *G. hispidum*, *G. doloresi* and *G. nipponicum* from all cases with available individual data (N = 310). **(A)** *G. spinigerum* + *G. binucleatum* (N = 255 [*G.s.* 248, *G.b.* 7]); **(B)** *G. hispidum* + *G. doloresi* + *G. nipponicum* (N = 55 [*G.h.* 9, *G.d.* 37, *G.n.* 9]) [Note: *G. malaysiae* was not included, since data were restricted to a single case, see Table 5]; [Body template adapted from an open source clipart (Link to source: https://openclipart.org/detail/314881/human-male-and-female-body-line-art)].

Several species-specific differences in lesion morphology and anatomical patterns are described in the literature, but these narrative descriptions originate primarily from clinical experts, and the available data is rather sparse. CLM lesions due to *G. spinigerum* and *G. binucleatum* are described to present identically as intermittent migratory swelling (less frequent as serpiginous dermal tracks) primarily located on the peripheral extremities, the face and the head with symptoms persisting untreated over >1–4 years [6]. In contrast, CLM lesions due to *G. hispidum*, *G. nipponicum* and *G. doloresi* are described to mainly present as serpiginous dermal tracks (less frequently as migratory swelling) primarily located on the central chest, abdomen and the back with symptoms persisting untreated over <2–3 months [6]. To verify these species-specific differences described, we analyzed the reviewed cases respectively (Table 4, Figs 9 and 10). The available data do not allow for an assessment of the individual species-specific duration of symptoms and the limited number of non-*spinigerum* CLM cases restricts the strength of the analysis. However, our species-specific analysis of the anatomical pattern and lesion morphology nevertheless corresponds with the literature (Table 4, Figs 9 and 10).

**Table 4:** Analysis of the reported larval migration pattern of the different human pathogenic *Gnathostoma* spp. in CLM patients (N = 261).

	Total number of migrating larvae (N)	Superficial tissue migration¹ N (%)	Deep tissue migration² N (%)	Superficial and deep tissue migration¹,² N (%)
***G. spinigerum***	198	28 (14.1)	160 (80.8)	10 (5.1)
***G. doloresi***	37	18 (48.7)	8 (21.6)	11 (29.7)
***G. binucleatum***	7	2 (28.6)	5 (71.4)	0 (0)
***G. hispidum***	9	7 (77.8)	1 (11.1)	1 (11.1)
***G. nipponicum***	9	9 (100)	0 (0)	0 (0)
***G. malaysiae***	1	1 (100)	0 (0)	0 (0)

¹ Superficial: serpiginous dermal tracks; ² Deep: subcutaneous swellings.

CLM, cutaneous larva migrans.

Organ invasive larval migration beyond the tegument is seen in 16.4% of the cases (either consecutively following CLM or as primary manifestation; Table 5). These cases present with NLM (8.2%), either in the form of primary NLM (7.6%) or as consecutive NLM (0.6%) following CLM or OLM (Table 5), as OLM (3.5%), or as VLM (2.3%).

**Table 5:** Frequency of clinical syndromes and their combinations caused by human pathogenic *Gnathostoma* species in the reviewed literature (N = 2101).

Clinical manifestation		Number of reported cases	
**Mono-syndromal**	**Multi-syndromal**	**N**	**%**
CLM		1757	83.6
	CLM ➔ OLM	21	1.0
	CLM ➔ VLM	14	0.7
	CLM ➔ NLM	9	0.4
	CLM ➔ VLM ➔ NLM	1	0.05
	CLM ➔ NLM ➔ VLM	1	0.05
NLM		161	7.7
	NLM ➔ CLM	4	0.2
	NLM ➔ VLM	1	0.05
	NLM ➔ OLM ➔ CLM	1	0.05
OLM		73	3.5
	OLM ➔ NLM	2	0.1
VLM		49	2.3
	VLM ➔ CLM	7	0.3

CLM, cutaneous larva migrans; NLM, neural larva migrans; OLM, ocular larva migrans; VLM, visceral larva migrans.

➔: Progression from one clinical syndrome to another during the course of infection.

**Fig 11:**
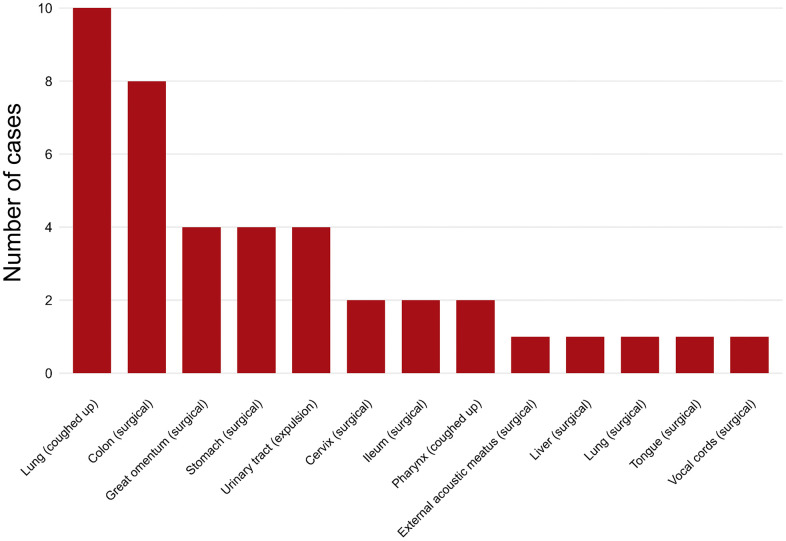
Reported anatomic location of gnathostomal VLM and how the larvae were retrieved (N = 41).

In the cases of VLM, any organ may be affected by migrating larvae with a diverse range of reported retrieval sites. Larvae are either removed surgically or detected by the patient in sputum or in urine (Fig 11). The high proportion of cases in which patients reported the expulsion of larvae in sputum and urine can be explained by the fact that the presence of larvae in sputum and urine is more likely to be noticed than, for example, the expulsion of larvae in stool. Given that surgical removal of larvae from the intestinal tract is reported relatively frequently, it can be assumed that the latter does occur but goes unnoticed. Looking at the reported sites where larvae are frequently surgically removed, the prominence and immediate proximity of the frequently reported organs (colon, stomach, greater omentum; Fig 11) plausibly reflects the natural anatomical route of larvae after peroral infection. Unlike in NLM and OLM, viscerally migrating larvae are considerably less likely to cause significant pathology, consequently leading to surgical removal and diagnosis. Considering that in all cases presenting with CLM, the larvae must have previously passed through visceral tissue, it appears plausible to assume that the VLM cases which do not progress to consecutive CLM are in a lot of cases not diagnosed.

**Fig 12:**
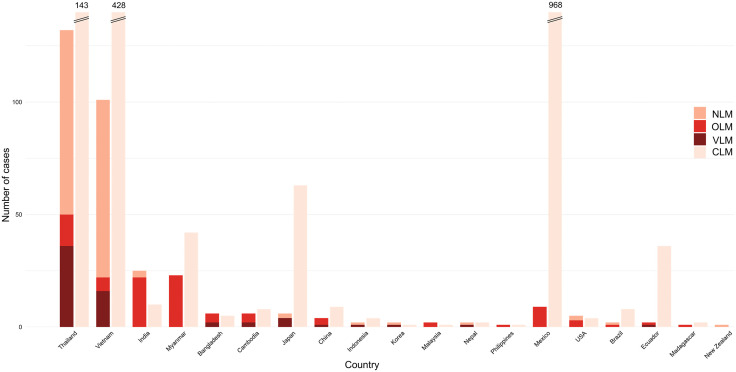
Reported NLM, OLM, VLM and CLM cases in different countries in the reviewed literature (N_cases_ = 2067). CLM, cutaneous larva migrans; NLM, neural larva migrans; OLM, ocular larva migrans; VLM, visceral larva migrans.

With regards to NLM and OLM, the question arises as to whether the reported frequencies of these clinical syndromes are due to actual neurotropism of the parasite or simply to the fact that invasion of these anatomical structures leads to more severe symptoms and thus are more likely to be diagnosed. Gnathostomal NLM is predominantly reported in the *G. spinigerum* endemic areas of Southeast Asia and rarely elsewhere (Fig 12). As discussed above, CLM caused by *G. spinigerum* and CLM caused by *G. binucleatum* are reported to exhibit identical patterns in terms of anatomical predilection and the tendency to show predominantly deeper rather than superficial tissue migration. The higher tendency towards deeper tissue migration would be consistent with the tendency to cause more NLM (compared to *Gnathostoma* spp. showing more superficial tissue migration). It is therefore striking that NLM is reported almost exclusively in the *G. spinigerum* endemic areas of Southeast Asia, while it is virtually unknown in the *G. binucleatum* endemic areas of Latin America (to date, only three autochthonous NLM cases on the American continent have been described, two in the USA [60,61] and one in Brazil [62]). Thus, a species-specific neurotropism of *G. spinigerum* appears possible, but remains to be proven.

**Fig 13:**
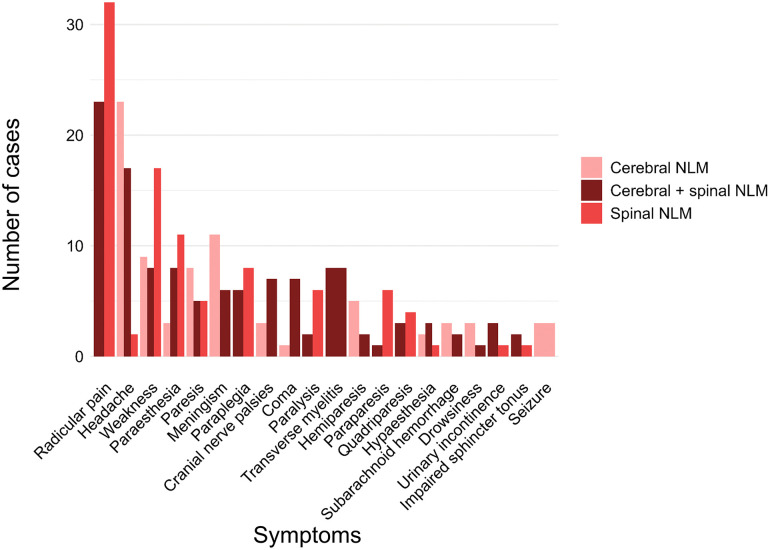
The top 20 reported symptoms in gnathostomal NLM categorized by NLM subtype (N_symptoms_ = 282). NLM, neural larva migrans.

NLM cases can roughly be split into three clinical forms: a cerebral, a spinal and a combined form, depending on the location of the neuronal damage caused by the migrating larvae. Findings in the cerebral form mainly include headaches, meningism, cranial nerve palsies and subarachnoid hemorrhages, while the spinal form is mainly associated with radicular pain, paresthesia, paresis or plegia, and the combined form exhibits findings from both forms (Fig 13). The *Gnathostoma* larva reaches the central nervous system via tissue migration along nerve roots, causing radiculomyelitis. The larva then ascends along the spinal nerves and vessels into the brain, causing direct mechanical damage to the tissue. This can be seen as hemorrhagic tracts in neurologic imaging and often leads to subarachnoid hemorrhage with red blood cells (RBC) in the cerebrospinal fluid (CSF) [63–65]. In Southeast Asia, where most gnathostomal NLM cases are reported, the main differential diagnosis is cerebral angiostrongyliasis due to the neurotropic neuroinvasive larvae of the nematode *Angiostrongylus cantonensis*. Since *A. cantonensis* larvae are significantly smaller (450–600 x 20–30 µm), enter the brain via the bloodstream, and have a more limited lifespan (2–8 weeks), the clinical picture and the prognosis differ considerably. Cerebral angiostrongyliasis primarily presents as eosinophilic meningitis, radicular symptoms are uncommon, migratory tracks are typically not visible on imaging, the course is usually mild and self-limiting, and subarachnoid hemorrhage and sequelae are rare [65,66].

In gnathostomal OLM, all anatomical parts of the eye may be affected (Table 6), but the reported frequency of affected ocular structures suggests that the larvae mostly enter the eye from the anterior periorbital soft tissue. This is also suggested by case reports describing pain and swellings around the eye before the onset of ocular symptoms [61,67–69] and matches the anatomic CLM distribution pattern of *G. spinigerum* and *G. binucleatum* showing a predilection for the face and particularly the periocular region (Fig 10). This may also explain why to date, *G. spinigerum* and *G. binucleatum* are the only *Gnathostoma* spp. reported to cause OLM. However, in cases where there were no previous CLM lesions on the face but bleeding in the retinal vessels, retinal holes or fundus scars were observed, eye invasion may also have occurred via the bloodstream [70–73]. Compared to NLM, the occurrence of OLM appears to be more evenly distributed in the endemic areas, with the exception of a noticeable clustering of OLM cases reported from India and more recently from Myanmar [74]. This is particularly strange because, in comparison, CLM is very rarely reported from these countries (Fig 12). The reported symptoms and pathologies associated with gnathostomal OLM result from the invasion and migration of the larvae and are diverse but non-specific (Table 7).

**Table 6:** Reported anatomical ocular structures affected in gnathostomal OLM cases (N_larvae_ = 78).

Reported anatomical structures affected in gnathostomal OLM^1^	Reported frequency N (%)
Anterior chamber	50 (61.7)
Vitreous body	13 (16.1)
Iris	8 (9.9)
Retina	4 (4.9)
Posterior chamber	3 (3.7)
Cornea	2 (2.5)
Lens	1 (1.2)

OLM, ocular larva migrans.

^1^In individual cases multiple affected ocular structures have been mentioned over the course of infection, thus their sums exceeding the count of N_larvae_.

**Table 7:** Top 20 symptoms in gnathostomal OLM cases (N_symptoms_ = 283).

Reported symptoms in gnathostomal OLM	Reported frequency N (%)
Reduced/blurred vision	63 (22.3)
Ocular pain	46 (16.3)
Ocular redness	37 (13.1)
Ocular swelling	31 (11.0)
Increased intraocular pressure	20 (7.1)
Uveitis	18 (6.4)
Bleeding	12 (4.2)
Photophobia	9 (3.2)
Tearing	7 (2.5)
Chemosis	6 (2.1)
Panuveitis	6 (2.1)
Floater	5 (1.8)
Posterior synechia	5 (1.8)
Ciliary congestion	3 (1.0)
Hypopyon	3 (1.0)
Ptosis	3 (1.0)
Conjunctivitis	3 (1.0)
Congestion	2 (0.7)
Itching	2 (0.7)
Vitreous haze	2 (0.7)

OLM, ocular larva migrans.

Like other tissue-invasive helminth infections, gnathostomiasis is often accompanied by blood eosinophilia. Although no universal threshold value exists to define eosinophilia, a widely accepted cut-off is ≥ 500 eosinophils/μl (≥0.5 × 10⁹ eosinophils/l) [75]. The considerably lower frequency of peripheral eosinophilia in OLM compared to CLM, VLM and NLM is likely explained by the fact that the ocular space is an immunological sanctuary containing very few immune cells (even less than in the CNS) and being separated from the rest of the body by the blood-retina barrier [76]. Thus, once inside the eye, the systemic eosinophilic stimulation caused by the tissue migration of the larva(e) apparently abates. Although the CNS is also considered an immune privileged site [77], we interestingly found that this apparently does not influence the systemic eosinophilic reaction (Table 8).

**Table 8:** Reported blood eosinophil count according to gnathostomal syndrome (N_cases_ = 2030).

	CLM	NLM	OLM	VLM
Number of cases with reported eosinophil data	191	36	13	17
Eosinophil count total [x10⁹/L] median (range)	746 (47–20,925)	1,798 (296–73,344)	588 (302–4,775)	1,493 (87–76,821)
Eosinophil count in % median (range)	14.5 (0.8–75.0)	13.0 (2.0–96.0)	7.5 (4.0–25.0)	19.3 (2.8–87.0)
% of cases with an eosinophil count of ≥500/µl	68.1	83	30.8	70.6

CLM, cutaneous larva migrans; NLM, neural larva migrans; OLM, ocular larva migrans; VLM, visceral larva migrans.

Definitive diagnosis of gnathostomiasis relies on identification of *Gnathostoma* larvae in biopsy specimen or when they spontaneously emerge from CLM lesions. Since this is limited to a relatively small proportion of cases, serology remains the most commonly reported diagnostic method (Table 9). Since in most of the publications reviewed the data on the serological tests used were largely insufficient or missing, we refrained from attempting any analysis. A detailed summary of *gnathostoma*-specific serological assays is beyond the scope of this review and can be found elsewhere [6], but we would like to highlight some core aspects. In the absence of commercially available serological assays, serological testing remains limited and largely restricted to academic institutions offering diagnostic laboratory services (e.g., the Department of Helminthology at Mahidol University, Bangkok, Thailand or the Diagnostic Centre at the Swiss Tropical and Public Health Institute, Basel, Switzerland). To date, most serological assays rely on preparations of crude antigen of AL3, harvested from second intermediate hosts. Cross-reactivity of anti-*Gnathostoma* antibodies in serological assays is observed [78], but not necessarily present. Thus, anti-*Gnathostoma* antibodies in a patient’s blood may not always be detectable by an assay prepared from crude antigen of a *Gnathostoma* species not identical to the one infecting the patient [40]. Serological assays based on recombinant *Gnathostoma* antigens as well as a point-of-care immunochromatographic assay have been developed and it is hoped that in the future such assays will become commercially available [79–81]. In general, however, it should always be borne in mind that serology is an indirect diagnostic method with incomplete sensitivity and specificity. Although it can support the diagnosis, it must always be interpreted with caution and in conjunction with epidemiological and clinical probability.

**Table 9:** Diagnostic methods used to diagnose gnathostomiasis cases in the reviewed literature with respective data (N_cases_ = 1356).

Applied diagnostic method		N_cases_*	%
Serology		1087	80.1
	ELISA	745	54.9
	Western blot	319	23.5
	Ouchterlony	7	0.6
	ICT	3	0.2
	DIGFA	1	0.07
	IFA	1	0.07
	Unspecified	11	0.8
Biopsy	Microscopy	256	18.9
	PCR	13	1.0

ELISA, enzyme-linked immunosorbent assay; ICT, immunochromatographic test; DIGFA, dot-immunogold filtration assay; IFA, indirect fluorescent assay.

* Since in some cases more than one diagnostic method has been applied, the number of cases surpasses the total number of cases reported.

For cases in which larvae can be obtained by biopsy, or larvae spontaneously emerge from CLM lesions, we compiled a diagnostic bench aid to facilitate identifying the species (S1 File).

Since in most cases the larvae cannot be removed manually nor do they spontaneously emerge from the CLM lesions, treatment with albendazole and/or ivermectin is the primary treatment method. The most used regimens are albendazole 2 x 400 mg/day over 21 days, ivermectin 0.2 mg/kg over 1–2 days or the combination of both (Table 10). When looking on the most used regimens, the summarized treatment success rates in the reviewed cases was highest for albendazole and ivermectin combination therapy (86.7%), followed by albendazole monotherapy (73.5%), and lowest with ivermectin monotherapy (56.7%) (Table 11).

**Table 10:** Reported anthelmintic CLM first line treatment regimens and their success rates in the reviewed gnathostomiasis cases (n_cases_ = 223).

First line treatment [treatment regimen] (N)	Cure N (%)	Failure N (%)
Albendazole [200–1200¹ mg/d x 2–28^2^ days] (98)	72 (73.5)	26 (26.5)
Ivermectin [1 day 0.2 mg/kg BW] (38)	20 (52.6)	18 (47.4)
Ivermectin [2 days 0.2 mg/kg BW] (17)	10 (58.8)	7 (41.2)
Ivermectin [3 days 0.2 mg/kg BW] (2)	1 (50)	1 (50)
Ivermectin [5 days 0.2 mg/kg BW] (1)	0 (0)	1 (100)
Ivermectin [unspecified] (6)	5 (83.3)	1 (16.7)
Mebendazole [300 mg/d x 3–5^3^ days] (9)	6 (66.7)	3 (33.3)
Thiabendazole [30 mg/kg BW x 5–17^4^ days] (2)	1 (50)	1 (50)
Diethylcarbamazine [150 mg/kg BW x 21 days] (3)	3 (100)	0 (0)
Albendazole [800–1200^5^ mg/d x?–21^6^ days] + Ivermectin [1 day 0.2 mg/kg BW] (42)	36 (85.7)	6 (14.3)
Albendazole [1200 mg/d x 7 days] + Ivermectin [2 days 0.2 mg/kg BW] (1)	?^7^	?^7^
Albendazole [unspecified] + Ivermectin [unspecified] (2)	2 (100)	0 (0)
Albendazole [? mg/d x 21 days] + Mebendazole [?] (1)	1 (100)	0 (0)
Albendazole [800 mg/d x 21 days] + Praziquantel [20 mg/kg BW/day x 1 day] (1)	1 (100)	0 (0)

BW, body weight.

**¹** Median daily dosage 800 mg

^**2**^ Median duration of treatment 21 days

^**3**^ Median duration of treatment 3 days

^**4**^ Median duration of treatment 11 days

^**5**^ Median daily dosage 1200 mg

^**6**^ Median duration of treatment 21 days

^**7**^ Patient lost to follow up

**Table 11:** Reported anthelmintic CLM treatment regimens and their success rates in the reviewed gnathostomiasis cases (n_cases_ = 223).

First line treatment			Second line treatment			Third line treatment			Further treatment
Regimen N	Cure N (%)	Failure N (%)	Regimen N	Cure N (%)	Failure N (%)	Regimen N	Cure N (%)	Failure N (%)	Regimen (outcome)
Albendazole (98)	72 (73.5)	26 (26.5)➔	➔ Albendazole (18)	11 (61.1)	7 (38.9)➔	➔ Albendazole (6)	5 (71.4)	1 (28.6)➔	➔ Albendazole (failure) ➔ Ivermectin (cure)
						➔ Ivermectin (1)	1 (100)	0 (0)	–
			➔ Ivermectin (6)	6 (100)	0 (0)	–	–	–	–
			➔ Thiabendazole (2)	0 (0)	2 (100)➔	➔ Albendazole (1)	0 (0)	1 (100)➔	➔ Albendazole (failure) ➔ Albendazole (failure) ➔ Ivermectin (cure)
						➔ Praziquantel (1)	0 (0)	1 (100)➔	➔ Ivermectin (failure) ➔ Albendazole (cure)
Ivermectin (64)	36 (56.2)	28 (43.8)➔	➔ Albendazole (21)	19 (90.5)	2 (9.5)➔	➔ Albendazole (1)	0 (0)	1 (100)➔	➔ Albendazole (cure)
						➔ Albendazole + Ivermectin (1)	1 (100)	0 (0)	–
			➔ Ivermectin (6)	4 (66.7)	2 (33.3)➔	➔ Ivermectin (2)	1 (50)	1 (50)➔	➔ Albendazole (failure) ➔ Albendazole (cure)
			➔ Albendazole + Ivermectin (1)	0 (0)	1 (100)➔	➔ Ivermectin (1)	0 (0)	1 (100)➔	➔ Ivermectin (failure) ➔ Ivermectin (failure) ➔ Ivermectin (failure) ➔ Ivermectin (cure)
Albendazole + Ivermectin (45)	39 (86.7)	6 (13.3)➔	➔ Albendazole (5)	5 (100)	0 (0)	–	–	–	–
			➔ Albendazole + Ivermectin (1)	1 (100)	0 (0)	–	–	–	–
Mebendazole (9)	6 (66.7)	3 (33.3)➔	➔ Thiabendazole (1)	1 (100)	0 (0)	–	–	–	–
			➔ Albendazole (2)	0 (0)	2 (100)➔	➔ Ivermectin (1)	0 (0)	1 (100)➔	➔ Ivermectin (failure) ➔ Ivermectin (failure) ➔ Ivermectin (failure)
						➔ Mebendazole (1)	0 (0)	1 (100)➔	➔ Mebendazole (failure) ➔ Albendazole (failure) ➔ Ivermectin (failure) ➔ Albendazole (failure) ➔ Albendazole (cure)
Thiabendazole (2)	1 (50)	1 (50)➔	➔ Thiabendazole (1)	1 (100)	0 (0)	–	–	–	–
Albendazole + Mebendazole (1)	1 (100)	0 (0)	–	–	–	–	–	–	–
Albendazole + Praziquantel (1)	1 (100)	0 (0)	–	–	–	–	–	–	–
Diethylcarbamazine (3)	3 (100)	0 (0)	–	–	–	–	–	–	–

To date, six prospective clinical studies have evaluated the efficacy of anthelminthic therapy in gnathostomiasis (Table 12). All the six trials were conducted in Thailand (thus, concerned *G. spinigerum*) and five of them evaluated the efficacy of albendazole or ivermectin against placebo or against each other. No studies on combination therapy have been conducted to date. Compared to our merged data, the treatment success rates in these studies were overall higher, but in line with our data higher for albendazole monotherapy (93–94.1%) [43,44,51] and lower for ivermectin monotherapy (50–95.2%). Combined usage of albendazole and ivermectin appears promising; however, the apparent benefits of this combination therapy should be interpreted with caution, as the available evidence is based almost exclusively on retrospective case reports and case series without prospective comparative evaluation. Combination therapy therefore requires further validation in prospective controlled studies to examine its efficacy and optimal use.

**Table 12:** Treatment success rates of different CLM treatment regimens in literature.

Author (year)	Type of study	Dosage	Patients (n)	Follow up time	Cure rate (%)	Failure rate (%)	Duration until lesion clearance in days; median (range)	Comment
Bussaratid V. et al. (2006) [49]	Prospective randomized placebo-controlled trial (IVM vs. placebo)	0.2 mg/kg IVM single dose	14	12 months	50^1^	50^1^	6 (6 – 6)	Patients sustaining relapses/treatment failure received ABZ 400 mg BID for 14d. Outcome of 2^nd^ line treatment not reported.
Bussaratid V. et al. (2005) [50]	Prospective single-arm observational study (IVM tolerability)	0.05 – 0.2 mg/kg IVM single dose	15	6 months	87^2^	13^2^	3.8 (1 – 6)	Patients sustaining relapses/treatment failure received ABZ 400 mg BID for 14d. Outcome of 2^nd^ line treatment not reported.
Kraivichian K. et al. (2004) [51]	Prospective randomized open-label trial (ABZ vs. IVM)	➔ 400 mg ABZ BID for 21d	14	4 months	93	7	6.8 (3 – 30)	1 Patient in ABZ group did not respond to the treatment. Cure after excisional skin biopsy containing AL3.
		➔ 0.2 mg/kg IVM single dose	17	4 months	76	24	6 (3 – 40)	Patients sustaining relapses/treatment failure received IVM (0.2 mg/kg) for 2 consecutive days. One IVM retreatment induced eruption of an AL3. Outcome of 2^nd^ line treatment: 100% cure. No statistically significant difference in cure rates between ABZ and IVM (p > 0.05). More flaring of cutaneous lesions (aching, itching, creeping, swelling) reported in IVM arm (58% vs 28%).
Nontasut P. et al. (2000) [44]	Prospective 2-armed cohort study (ABZ vs IVM)	➔ 400 mg ABZ BID for 21d	49	6 months	93.8	6.2	*≤ 7d*	Treatment induced eruption of 3 AL3.
		➔ 0.2 mg/kg IVM single dose	21	6 months	95.2	4.8	*≤ 7d*	Treatment induced eruption of 1 AL3.
Suntharasamai P. et al. (1992) [52]	Prospective randomized placebo-controlled trial (ABZ vs. placebo)	400 mg ABZ BID for 14d	41	12 months	No data^3^	No data^3^	5 (1 – 17)	Treatment induced eruption of 3 AL3^4^. Highly significant difference between ABZ and placebo in outward larva migration (p < 0.0001).
Kraivichian P. et al. (1992) [43]	Prospective randomized placebo-controlled trial (ABZ vs. placebo)	➔ 400 mg ABZ BID for 21d	49	6 months	93.9	6.1	6.4 (1 – 14)	Treatment induced eruption of 9 AL3 from 9 patients.
		➔ 400 mg ABZ OD for 21d	51	6 months	94.1	5.9	6.2 (1 – 12)	Treatment induced eruption of 13 AL3 from 12 patients.

IVM, ivermectin; ABZ, albendazole; BID, twice daily; OD, once daily.

^1^The authors mentioned a cure- and failure rate of 41.2%, counting with 17 patients, however, 3 patients were lost to follow up.

^2^The authors mentioned 30% relapses; however, 2 patients were lost to follow up before the first revisit, and 3 patients most likely did not have gnathostomiasis.

^3^The primary end point was larva retrieval, either by spontaneous outward migration of the worm through the patient’s skin or by minor excision.

^4^Author addendum - after the initial study another 273 cases of CLM were treated with 400 mg ABZ BID for 14 days with 17 treatment induced AL3 eruptions.

Retreatment and sometimes even multiple cycles of retreatment may be necessary in the case of treatment failure (Table 11). In the absence of evidence for a best retreatment regimen, the most frequently adopted strategy is changing to the alternative drug, extending the duration of treatment, or opting for combination therapy (Table 11). In some cases, anthelmintic treatment has been reported to lead to more superficial migration of *Gnathostoma* larvae with the possibility to extract the parasite from a small erupting papule [44,51] or even the spontaneous emergence of the larva [82]. This phenomenon of treatment induced larval outward migration/ emergence has mainly been described with albendazole [52], but similar observations were also reported with ivermectin [44,51,83], under interferon alpha therapy in a hepatitis C patient [84], and following praziquantel treatment [85], although the latter two drugs lack evidence regarding a plausible mode of action against *Gnathostoma* larvae and, thus remain debatable.

Besides anthelminthic treatment adjunctive corticosteroid treatment is used to control cerebral inflammation and edema in cases of NLM [46]. In the absence of evidence from clinical trials this approach is based on plausible theoretical considerations and the successful use of corticosteroids in other inflammatory CNS processes, including parasitic infections like, e.g., cerebral angiostrongyliasis, neurocysticercosis and neuroschistosomiasis [86].

Infrequently the use of other anthelmintic drugs (i.e., mebendazole, thiabendazole, diethylcarbamazine, praziquantel) in human gnathostomiasis has been reported (Table 10), but clinical studies evaluating their effectiveness are lacking. The effectiveness of other benzimidazole compounds such as mebendazole and thiabendazole appears plausible due to their identical mode of action to albendazole, but thiabendazole is no longer marketed and the inferior tissue penetration of mebendazole compared to albendazole argues against its use. The reported use of diethylcarbamazine (DEC) in three cases appears to be based on an anticipated pan-nematode activity of the compound, which is primarily used to treat filarial infections [87] whereas the effectiveness of praziquantel against the nematode *Gnathostoma* is not plausible, since the compound shows a trematode-specific mode of action [87]. In the only cases reported to have received albendazole plus praziquantel (Table 10), gnathostomiasis was initially not suspected (the case was a tourist returning from Botswana), the antiparasitic treatment empirically chosen, and the diagnosis only latter established after the larva emerged from the skin [24].

We found reported cure rates of gnathostomal CLM and VLM of 94.2% and 100%, respectively and no sequelae reported in these cases (Table 13). However, in 5.8% of the reviewed gnathostomal CLM cases failure of anthelminthic treatment was reported, leading to recurrent/relapsing CLM lesions. The true recurrence/relapse rate of human gnathostomiasis is difficult to assess, since systematic long-term follow-up data is limited and in endemic areas distinguishing treatment failure from reinfection is problematic. In this context, a French cohort study in particular has contributed interesting data: 13 French travellers who had been infected in Southeast Asia (n = 11) and Mexico (n = 2) were followed up for a median of 15 months (6–49 months) after favourably responding to first-line treatment with albendazole (n = 12) or ivermectin (n = 1) [47]. Eight of the 13 patients (61.5%) sustained a total of 13 relapses (1–4 relapses per patient). The median interval between initial treatment and the first relapse was 2 months (range 1–7 months), the interval between successive relapses was 5–30 months [47]. The relapse rate of 54.2% we found among the reviewed CLM cases confirms that treatment failures are overall frequent. Nevertheless, despite the high frequency of CLM relapses, cases unresponsive to medical treatment were only reported in 5.8% of the reviewed cases and therefore relatively rare. The very low number of VLM and NLM cases compared to CLM does not allow to answer the question whether differences regarding the relapse rate exist between the different gnathostomal syndromes (Table 13). Due to the overall limited number of non-*spinigerum* infections, an analysis regarding potential differences in treatment success and failure rates between the different *Gnathostoma* species was not feasible. In patients who suffer multiple relapses and undergo repeated treatment, it ultimately remains unclear whether the therapy was finally effective or whether the absence of further relapses was due to the natural death of the larvae. Thus, the effectiveness of medical treatment may be overestimated. More recently, the observation of morphological changes in the tegumental structures of *G. spinigerum* larvae that survived albendazole treatment has led to the speculation that the parasite may possibly be capable of developing adaptive tolerance/resistance to the drug [88].

**Table 13:** Reported frequency of a relapsing course despite and final outcome of medical treatment of the reviewed gnathostomiasis cases with available individual data (N_cases_ = 136; N_cases_ = 358).

Clinical syndrome	Relapsing course despite medical treatment		Final outcome of medical treatment				
	N_cases_ with respective data	Relapsing cases^1^ N (%)	N_cases_ with respective data	Cure N (%)	Unresponsive to medical treatment^2^ N (%)	Sequelae N (%)	Death N (%)
CLM^3^	107	58 (54.2)	224	211 (94.2)	13 (5.8)	0 (0)	0 (0)
VLM	2	11 (50)	22	22 (100)	0 (0)	0 (0)	0 (0)
NLM	9	4 (44.4)	56	19 (33.9)	0 (0)	18 (32.2)^4^	19 (33.9)^5^
OLM	18	0 (0)	56	27 (48.2)	0 (0)	29 (51.8)^6^	0

CLM, cutaneous larva migrans; VLM, visceral larva migrans; NLM, neural larva migrans; OLM, ocular larva migrans.

1 Def.: Symptoms subside following medical treatment but reappear after a symptom-free period.

2 Def.: Medical treatment has no effect on the symptoms.

3 Cases of CLM progressing to NLM (n = 26) or OLM (n = 24) are included in the NLM and OLM data below.

4 Coma (n = 2); hemiparesis/hemisyndrome (n = 6); cranial nerve palsy (n = 2); memory problems (n = 2); aphasia (n = 1); paraplegia (n = 2); paraparesis (n = 1); spinal cord atrophy (n = 1); weakness (n = 1); anal sphincter dysfunction (n = 1); bladder dysfunction (n = 1); unspecified neurologic deficit (n = 4).*

5 Cerebral hemorrhage (n = 12); brain stem damage (n = 4); secondary infectious complications during hospitalization (n = 2).*

6 Reduced vision (n = 28); cataract (n = 2); unilateral blindness (n = 2); optic atrophy (n = 2).

* Since in individual cases several sequelae were concomitantly present, the sums of sequelae surpass the number of cases.

In gnathostomal OLM, the lack of reports of treatment failure (Table 13) and the high rate of sequelae (in 53.7% of cases) is not surprising, given that surgical removal of the larvae is the treatment of choice and defect healing of the highly sensitive ocular structures invaded by the larvae is to be expected.

Not surprisingly, the worst outcome is reported in gnathostomal NLM, with roughly one third of patients fully recovering, one third suffering from sequelae, and one third dying of the infection (Table 13).

We acknowledge that our systematic review has three main limitations: Firstly, the fact that our literature search may have not included all publications reporting on cases and studies of gnathostomiasis, particularly those in Thai or Japanese. As Thailand and Japan are high endemic regions, our language restriction may have influenced the analyses. Secondly, publication bias is an important limiting factor, as case reports and case series are more likely to describe more severe or unusual presentations. This may have resulted in overrepresentation of clinically striking manifestations and may have influenced estimates of disease severity, syndrome frequency, and treatment outcomes. Also the analyses on geographic distribution, syndrome frequency, and treatment patterns may have been influenced by the language restriction as well as publication bias. Thirdly, the already mentioned fact that the number of non-*spinigerum* cases was overall relatively small, limiting the species-specific analysis. Nevertheless, we hope that our analysis provides clinicians with a helpful overview of the clinically relevant aspects of human gnathostomiasis.

## Supporting information

S1 File Diagnostic bench aid. (DOCX)

S1 Text Search terms used. (DOCX)

S2 Text Systematic review protocol. (DOCX)

S3 Text PRISMA checklist. (DOCX)

S1 Table Data extraction sheet. (DOCX)

S2 Table Master table of raw data. (XLSX)

S3 Table Reference list of included publications. (XLSX)

S1 Fig Westernblot original photo. (PDF)
