# Correction: Human gnathostomiasis – A systematic review and analysis of the literature

**DOI:** 10.1371/journal.pntd.0014735

**Published:** 2026-09-18

**Authors:** The PLOS Neglected Tropical Diseases Staff

## Notice of Republication

This article was republished on September 2, 2026, to replace Supplementary Figure 1 for which the wrong version had been uploaded. The publisher apologizes for the errors. Please download this article again to view the correct version.
