# Supplementary material for: Human gnathostomiasis – A systematic review and analysis of the literature
Source: PLoS Negl Trop Dis. 2026 Jul 31;20(7):e0014546. doi: 10.1371/journal.pntd.0014546 (PMC13426996; doi:10.1371/journal.pntd.0014546)
Supplement: S1 File — (DOCX) [file pntd.0014546.s001.docx]

1. **Diagnostic morphological characteristics helpful for the microscopic identification of advanced third stage larvae (AL3) of human pathogenic *Gnathostoma* spp.**

The diagnostic characteristics of advanced third stage larva (AL3) of human pathogenic *Gnathostoma* spp. include (i) the number of head bulb hooklet rows and the number of hooklets per row (Fig A, Table A), (ii) the presence of cuticular spines covering the larva’s body (Fig B), (iii) the presence of large, cavernous lateral chords (Fig C), and (iv) some *Gnathostoma* spp. can be differentiated from each other on the basis of the morphologic features of their intestinal epithelial cells (Table A, Fig D).

**Fig A Microscopic head bulb morphology of advanced third stage larvae (AL3) of human pathogenic *Gnathostoma* spp.**


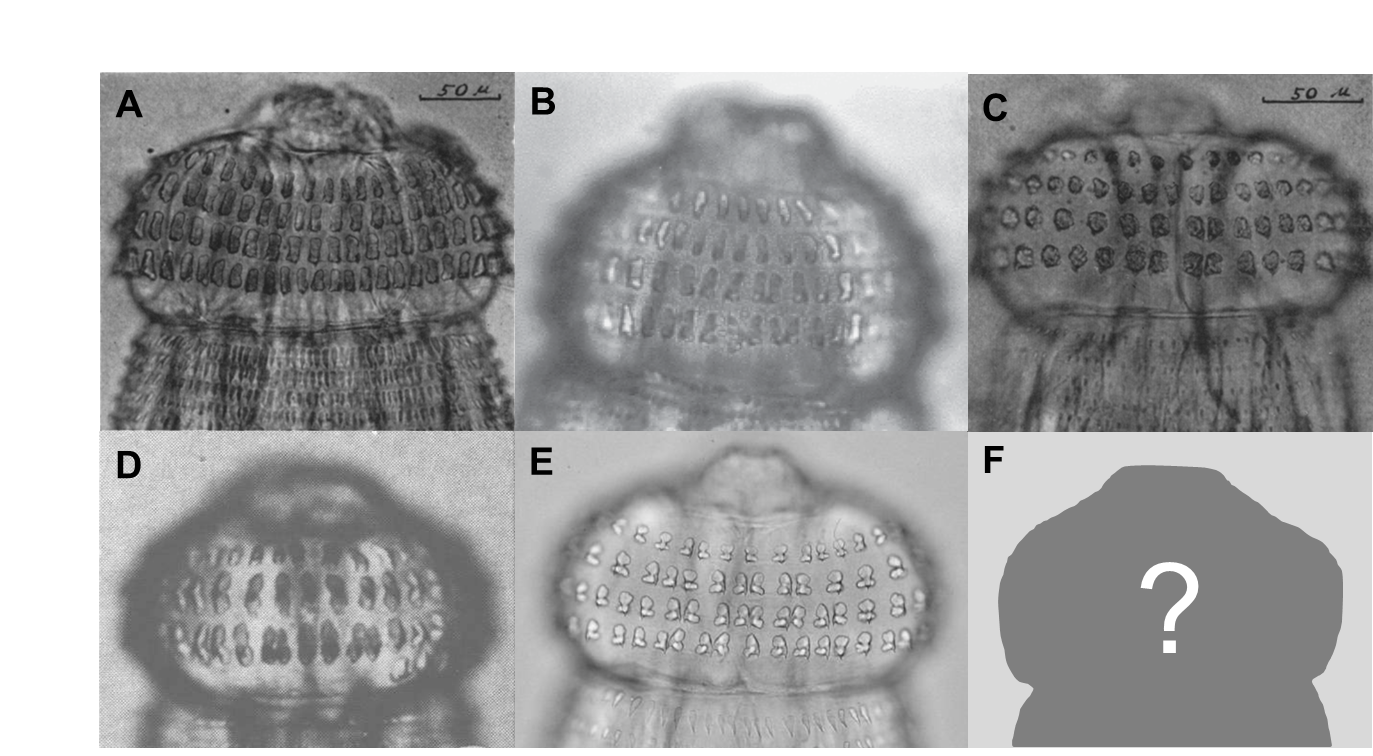


Head bulb of advanced third stage larvae (AL3) (A) *G. spinigerum*; (B) *G. binucleatum*; (C) *G. doloresi*; (D) *G. nipponicum*; (E) *G. hispidum*; (F) *G. malaysiae* [no image available]

(Image source: (A), (C) [1] (B) [2] (D) [3] (E) [4]).

Note the morphological differences of the basal part of hooklets of each species:

*G. spinigerum* and *G. binucleatum*: "oblong"; *G. doloresi*: "pebblestone"; *G. nipponicum* and *G. hispidum*: "irregular oblong".

**Table A Morphological characteristics of human pathogenic *Gnathostoma* spp. advanced third stage larvae (AL3).**

| ***Gnathostoma* spp.** | **Body length of advanced third stage larva (AL3) in mm** | **Number of head bulb hooklet rows** | **Number of hooklets per head bulb row** | **Morphology and distribution of body cuticular spines** | **Morphology of intestinal epithelial cells; No. of intestinal epithelial cells; No. of nuclei / intestinal epithelial cell** |
| --- | --- | --- | --- | --- | --- |
| *G. spinigerum* | 1.6 – 5.5 | 4 | 1: 40–44  2: 43–47  3: 45–50  4: 49–52 | Broad and short three-toothed Spines in first half, posterior 1/3 with very few spines | Columnar;  21–29;  3–7 |
| *G. binucleatum* | 1.5 – 2.5 | 4 | 1: 34–42  2: 39–46  3: 41–47  4: 42–52 | Whole body | Columnar;  17–25;  2–7 |
| *G. doloresi* | 0.7 – 6.3 | 4 | 1: 38–39  2: 37–39  3: 35–36  4: 35–38 | Broad and short three-toothed Spines in first half, posterior 1/3 with very few spines | Spherical (rounded or cuboidal);  18–28;  0–3 |
| *G. hispidum* | 2.0 – 2.6 | 4 | 1: 38–40  2: 40–44  3: 41–47  4: 44–48 | Spines over the whole body, increasing length of single-toothed spines towards the posterior end | Spherical (rounded or cuboidal);  19–31;  0–2 |
| *G. malaysiae* | 1.1 – 2.4 | 4 | 1: 44  2: 45  3: 49  4: 55 | Spines over the whole body, three-toothed in anterior half | Spherical (rounded or cuboidal);  20–26;  1 |
| *G. nipponicum* | 2.2 – 2.7 | 3 | 1: 29–37  2: 33–38  3: 36–43 | Three-toothed spines over the anterior body half, slightly convex margin, terminally increasing in breadth | Columnar;  10–14;  0–4 |

The data in the table are merged from various publications [1, 2, 4-8]; and lecture notes of Dr. Wichit Rojekittikhun, Dept. of Helminthology, Faculty of Tropical Medicine, Mahidol University, Bangkok, Thailand.

**Fig BCuticular spines covering the body of human pathogenic *Gnathostoma* spp. advanced third stage larvae (AL3).**

**
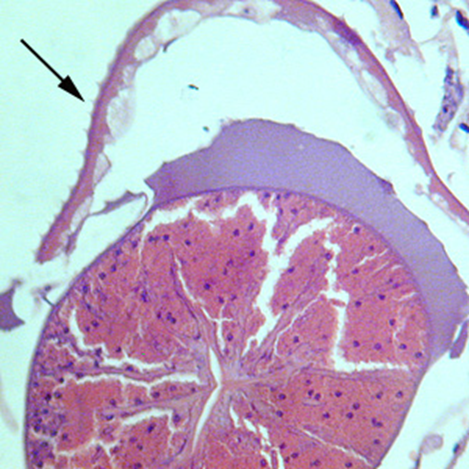
**

Cross section through a *Gnathostoma spinigerum* AL3 (at the level of the larva’s oesophagus, visible in the lower part of the picture): note the cuticular spines (arrow) covering the larva’s tegument / body surface (Hematoxylin-eosin (H&E)-stain). (Image source: https://www.cdc.gov/dpdx/gnathostomiasis/index.html).

**Fig C Morphological features of intestinal epithelial cells of advanced third stage larvae (AL3) of *Gnathostoma* spp. used for species identification.**

**
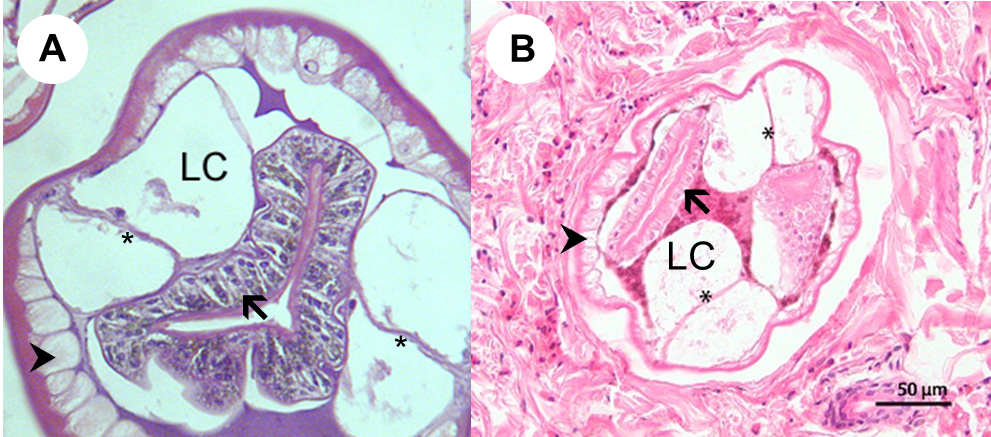
**

(A) HE-stained cross section of *Gnathostoma spinigerum* AL3 larva, characterized by the multinucleated columnar cells (with three to five nuclei per cell) of the intestinal epithelial cells (arrow). Note also the presence of pigmented granular material in the intestinal cells. (B) HE-stained cross section of *Gnathostoma hispidum* AL3 larva, characterized by the spherical (rounded to cuboidal) intestinal epithelial cells (arrow) with a single nucleus. Note also the typical genus-level larval features including low coelomyarian muscle cells (darts), and spacious lateral chords (LC) bisected by a lateral line (asterisks). (Image source: https://www.cdc.gov/dpdx/gnathostomiasis/index.html).

1. **Morphological characteristics of adult stage human pathogenic *Gnathostoma* spp.**

**Fig D Pictorial morphology of adult stage human pathogenic *Gnathostoma* spp. and their eggs.**

*
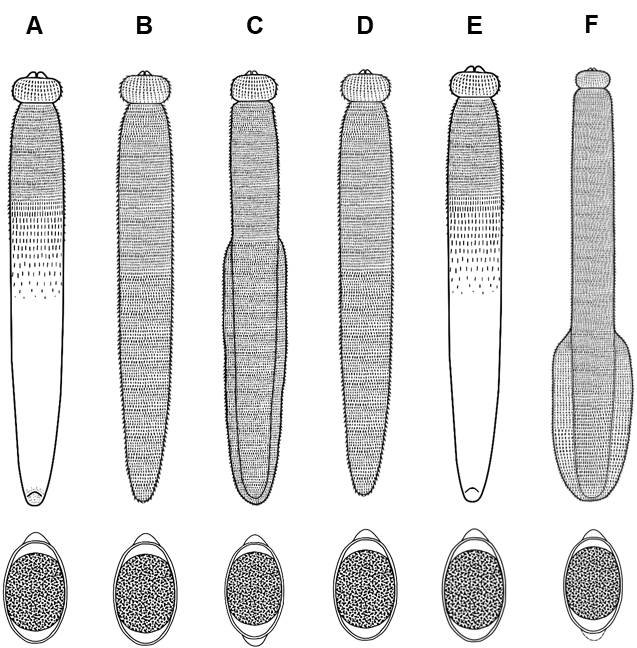
*

*(A) G. spinigerum*; (B) G. *binucleatum*; (C) *G. doloresi*; (D) *G. hispidum;* (E) *G. nipponicum*; (F) *G. malaysiae.*

**Table B Morphological characteristics of the adult stage of human pathogenic *Gnathostoma* spp.**

| ***Gnathostoma* spp.** | **Body length and diameter of adult stage parasite in mm** | | **Number of head bulb hooklet rows** |
| --- | --- | --- | --- |
|  | **Male** | **Female** |  |
| *G. spinigerum* | 12–40 x 1–3 | 13–55 x 1–3 | 7–9 |
| *G. binucleatum* | 15–22 x 0.9–1.2 | 21–25 x 1.1–1.4 | 8–10 |
| *G. doloresi* | 7–38 x 0.9–3 | 8–63 x 0.9–4.5 | 7–12 |
| *G. hispidum* | 19.7 x 1.7 | 26.2 – 2.3 | 9–12 |
| *G. malaysiae* | 13.6–20.4 x 0.8–1.8 | 28.6–33.5 x 1.6–2 | *no data available* |
| *G. nipponicum* | 10–27 | 17–45 | 7–8 |

(Data source: [5], lecture notes of Dr. Wichit Rojekittikhun, Dept. of Helminthology, Faculty of Tropical Medicine, Mahidol University, Bangkok, Thailand)

**Table C Morphological characteristics of the eggs of human pathogenic *Gnathostoma* spp.**

| ***Gnathostoma* spp.** | **Size of eggs in µm** | **Number of protruding mucus plugs on the egg poles** |
| --- | --- | --- |
| *G. spinigerum* | 55–79 x 34–42 | 1 |
| *G. binucleatum* | 58–68 x 37–39 | 1 |
| *G. doloresi* | 56–62 x 31–35 | 2 |
| *G. hispidum* | 65–69 x 31–35 | 1 |
| *G. malaysiae* | 65–69 x 34–38 | 1–2 |
| *G. nipponicum* | 69–76 x 39–45 | 1 |

(Data source: [5, 9], lecture notes of Dr. Wichit Rojekittikhun, Dept. of Helminthology, Faculty of Tropical Medicine, Mahidol University, Bangkok, Thailand)

**References**

1. Miyazaki I. On the genus Gnathostoma and human gnathostomiasis, with special reference to Japan. Exp Parasitol. 1960;9:338-70. doi: 10.1016/0014-4894(60)90040-0.

2. Lazo RF. Gnathostoma and gnathostomiasis in Ecuador. Southeast Asian J Trop Med Public Health. 2004;35(Suppl 1):92-6.

3. Ando K, Tokura H, Matsuoka H, Taylor D, Chinzei Y. life-cycle of gnathostoma-nipponicum yamaguti, 1941. J Helminthol. 1992;66(1):53-61. doi: 10.1017/S0022149X00012566. PubMed PMID: WOS:A1992HP51600008.

4. Cho S-H, Kim T-S, Kong Y, Na B-K, Sohn W-M. Larval Gnathostoma hispidum detected in the red banded odd-tooth snake, Dinodon rufozonatum rufozonatum, from China. Korean J Parasitol. 2007;45(3):191. doi: 10.3347/kjp.2007.45.3.191

5. Nawa Y, Maleewong W, Intapan PM, Díaz-Camacho SP. Gnathostoma. Biology of Foodborne Parasites: CRC Press; 2015. p. 405-26.

6. Nomura Y, Nagakura K, Kagei N, Tsutsumi Y, Araki K, Sugawara M. Gnathostomiasis possibly caused by Gnathostoma malaysiae. Tokai J Exp Clin Med. 2000;25(1):1-6. PubMed PMID: 33410598.

7. Han ET, Lee JH, Choi SY, Park JH, Shin EH, Chai JY. Surface ultrastructure of the advanced third-stage larvae of Gnathostoma nipponicum. J Parasitol. 2003;89(6):1245-8. doi: 10.1645/GE-3232RN. PubMed PMID: WOS:000187843500028.

8. Waikagul J, Chamacho SPD. Gnathostomiasis. In: Murrell KD, Fried B, editors. Food-Borne Parasitic Zoonoses: Fish and Plant-Borne Parasites. 112007. p. 235-61.

9. Miyazaki I, Dunn FL. Gnathostoma malaysiae sp. n. from rats on Tioman Island, Malaysia (Nematoda: Gnathostomidae). J Parasitol. 1965:382-4.
