# Supplementary material for: Human gnathostomiasis – A systematic review and analysis of the literature
Source: PLoS Negl Trop Dis. 2026 Jul 31;20(7):e0014546. doi: 10.1371/journal.pntd.0014546 (PMC13426996; doi:10.1371/journal.pntd.0014546)
Supplement: S1 Text — (DOCX) [file pntd.0014546.s002.docx]

Search terms and results

Search 1 (14.11.2024)

# Pubmed

| Platform | NCBI |
| --- | --- |
| Search date | 14.11.2024 |
| Search time frame | No restrictions |
| Search syntax | ((Gnathostoma [Mesh] OR Gnathostomiasis [Mesh]))  OR (Gnathostom* [tiab] OR “G.spinigerum” [tiab] OR “G.hispidum” [tiab] OR “G.doloresi” [tiab] OR “G.nipponicum” [tiab] OR “G.malaysiae” [tiab] OR “G.binucleatum” [tiab])  NOT (“Animals” [Mesh] NOT “Humans” [Mesh]) |
| Filter | None |
| Results | 818 |

# Web of science

| Platform | Clarivate |
| --- | --- |
| Search date | 14.11.2024 |
| Search time frame | No restrictions |
| Search syntax | TS=(gnathostom* OR "G.spinigerum" OR "G.hispidum" OR "G.doloresi" OR "G.nipponicum" OR "G.malaysiae" OR "G.binucleatum" OR "Gnathostoma infection") AND TS=(Humans OR Human) |
| Filter | None |
| Results | 1,534 |

# Scopus

| Platform | Elsevier |
| --- | --- |
| Search date | 14.11.2024 |
| Search time frame | No restrictions |
| Search syntax | TITLE-ABS-KEY ( gnathostom* OR "G.spinigerum" OR "G.hispidum" OR "G.doloresi" OR "G.nipponicum" OR "G.malaysiae" OR "G.binucleatum" ) AND TITLE-ABS-KEY ( human OR humans ) |
| Filter | None |
| Results | 998 |

# Embase

| Platform | Ovid |
| --- | --- |
| Search date | 14.11.2024 |
| Search time frame | No restrictions |
| Search syntax | (Gnathostom* or "G.spinigerum" or "G.hispidum" or "G.doloresi" or "G.nipponicum" or "G.malaysiae" or "G.binucleatum").ab,kw,sh,ti. |
| Filter | “Human” |
| Results | 707 |

# Medline

| Platform | Ovid |
| --- | --- |
| Search date | 14.11.2024 |
| Search time frame | No restrictions |
| Search syntax | (Gnathostom* or "G.spinigerum" or "G.hispidum" or "G.doloresi" or "G.nipponicum" or "G.malaysiae" or "G.binucleatum").ab,kw,sh,ti. |
| Filter | “Human” |
| Results | 637 |

# Cochrane

| Platform | Wiley |
| --- | --- |
| Search date | 14.11.2024 |
| Search time frame | No restrictions |
| Search syntax | (Gnathostom* OR "G.spinigerum" OR "G.hispidum" OR "G.doloresi" OR "G.nipponicum" OR "G.malaysiae" OR "G.binucleatum") AND (Humans OR Human) |
| Filter | None |
| Results | 8 |

# CINAHL

| Platform | EBSCOhost |
| --- | --- |
| Search date | 14.11.2024 |
| Search time frame | No restrictions |
| Search syntax | TI (Gnathostom*) OR AB (Gnathostom*) OR MW (Gnathostom*) OR SU (Gnathostom*) NOT (MH Animals NOT Humans) |
| Filter | None |
| Results | 51 |

Search 2 (20.08.2025)

# Pubmed

| Platform | NCBI |
| --- | --- |
| Search date | 20.08.2025 |
| Search time frame | No restrictions |
| Search syntax | ((Gnathostoma [Mesh] OR Gnathostomiasis [Mesh]))  OR (Gnathostom* [tiab] OR “G.spinigerum” [tiab] OR “G.hispidum” [tiab] OR “G.doloresi” [tiab] OR “G.nipponicum” [tiab] OR “G.malaysiae” [tiab] OR “G.binucleatum” [tiab])  NOT (“Animals” [Mesh] NOT “Humans” [Mesh]) |
| Filter | None |
| Results | 838 (+20) |

# Web of science

| Platform | Clarivate |
| --- | --- |
| Search date | 20.08.2025 |
| Search time frame | No restrictions |
| Search syntax | TS=(gnathostom* OR "G.spinigerum" OR "G.hispidum" OR "G.doloresi" OR "G.nipponicum" OR "G.malaysiae" OR "G.binucleatum" OR "Gnathostoma infection") AND TS=(Humans OR Human) |
| Filter | None |
| Results | 1554 (+20) |

# Scopus

| Platform | Elsevier |
| --- | --- |
| Search date | 20.08.2025 |
| Search time frame | No restrictions |
| Search syntax | TITLE-ABS-KEY ( gnathostom* OR "G.spinigerum" OR "G.hispidum" OR "G.doloresi" OR "G.nipponicum" OR "G.malaysiae" OR "G.binucleatum" ) AND TITLE-ABS-KEY ( human OR humans ) |
| Filter | None |
| Results | 1016 (+18) |

# Embase

| Platform | Ovid |
| --- | --- |
| Search date | 20.08.2025 |
| Search time frame | No restrictions |
| Search syntax | (Gnathostom* or "G.spinigerum" or "G.hispidum" or "G.doloresi" or "G.nipponicum" or "G.malaysiae" or "G.binucleatum").ab,kw,sh,ti. |
| Filter | “Human” |
| Results | 716 (+9) |

# Medline

| Platform | Ovid |
| --- | --- |
| Search date | 20.08.2025 |
| Search time frame | No restrictions |
| Search syntax | (Gnathostom* or "G.spinigerum" or "G.hispidum" or "G.doloresi" or "G.nipponicum" or "G.malaysiae" or "G.binucleatum").ab,kw,sh,ti. |
| Filter | “Human” |
| Results | 645 (+8) |

# Cochrane

| Platform | Wiley |
| --- | --- |
| Search date | 20.08.2025 |
| Search time frame | No restrictions |
| Search syntax | (Gnathostom* OR "G.spinigerum" OR "G.hispidum" OR "G.doloresi" OR "G.nipponicum" OR "G.malaysiae" OR "G.binucleatum") AND (Humans OR Human) |
| Filter | None |
| Results | 8 (+0) |

# CINAHL

| Platform | EBSCOhost |
| --- | --- |
| Search date | 20.08.2025 |
| Search time frame | No restrictions |
| Search syntax | TI (Gnathostom*) OR AB (Gnathostom*) OR MW (Gnathostom*) OR SU (Gnathostom*) NOT (MH Animals NOT Humans) |
| Filter | None |
| Results | 51 (+0) |
