# Supplementary material for: Human gnathostomiasis – A systematic review and analysis of the literature
Source: PLoS Negl Trop Dis. 2026 Jul 31;20(7):e0014546. doi: 10.1371/journal.pntd.0014546 (PMC13426996; doi:10.1371/journal.pntd.0014546)
Supplement: S2 Text — (DOCX) [file pntd.0014546.s003.docx]

Systematic review protocol

Table of contents

[Review title 2](#_Toc182374936)

[Timescale 2](#_Toc182374937)

[Review team details 2](#_Toc182374938)

[Review methods 3](#_Toc182374939)

[Review questions 3](#_Toc182374940)

[Searches 3](#_Toc182374941)

[Condition being studied 3](#_Toc182374942)

[Population 3](#_Toc182374943)

[Intervention 3](#_Toc182374944)

[Comparison/Control 4](#_Toc182374945)

[Types of study to be included initially 4](#_Toc182374946)

[Primary outcome 4](#_Toc182374947)

[Secondary outcome 4](#_Toc182374948)

[Measures of effect 4](#_Toc182374949)

[Selection strategy 4](#_Toc182374950)

[Data extraction 4](#_Toc182374951)

[Risk of bias (quality) assessment 7](#_Toc182374952)

[Strategy for data synthesis 7](#_Toc182374953)

[Analysis of subgroups or subsets 8](#_Toc182374954)

[Additional Information 8](#_Toc182374955)

# Review title

Human Gnathostomiasis – A systematic review of the literature

# Timescale

Anticipated Start date: 18 November 2024

Anticipated Completion date: 31 December 2025

# Review team details

Named Contact: Simon Frey

Named contact email: simon.frey@swisstph.ch

Organisational affiliation of the review:

- Swiss Tropical and Public Health Institute, Basel, Switzerland.
- University of Basel, Basel, Switzerland.

Team Members and their Organisational affiliation

- Simon Frey:
  - Swiss Tropical and Public Health Institute, Basel, Switzerland.
  - University of Basel, Basel, Switzerland.
- Andreas Neumayr:
  - Swiss Tropical and Public Health Institute, Basel, Switzerland.
  - University of Basel, Basel, Switzerland.
  - Department of Public Health and Tropical Medicine, College of Public Health, Medical and Veterinary Sciences, James Cook University, Queensland, Australia.
- Esther Kuenzli:
  - Swiss Tropical and Public Health Institute, Basel, Switzerland.
  - University of Basel, Basel, Switzerland.

Reviewer roles

- Primary reviewers: Simon Frey

Secondary reviewers: Andreas Neumayr, Esther Kuenzli

Quality assessors: Andreas Neumayr, Esther Kuenzli

Funding: Self-financed, no external funding

Conflicts of interest/ Competing interest: The authors declare that they have no known conflicts of interest.

Type and method of review: Systematic Review, Health area: Infections and infestations

Language: English

Country: Switzerland

Keywords: systematic review, human gnathostomiasis, *Gnathostoma*, *G. spinigerum, G. binucleatum, G. hsipidum, G. nipponicum, G. malaysiae, G. doloresi*

Current review status: ongoing

# Review methods

## Review questions

- What is the epidemiology and geographical occurrence of human gnathostomiasis?
- What are associated risk factors in the acquisition of human gnathostomiasis?
- What are the clinical presentation and laboratory findings of human gnathostomiasis?
- What anthelmintic treatment strategies are used against human gnathostomiasis?

## Searches

An electronic bibliographic database search will be performed on the following platforms: PubMed, Embase, Medline, CINAHL, Scopus, Cochrane and Web of Science Core Collection.

The search strategy will include the following terms for PubMed:

((Gnathostoma [Mesh] OR Gnathostomiasis [Mesh]))

OR (Gnathostom* [tiab] OR “G.spinigerum” [tiab] OR “G.hispidum” [tiab] OR “G.doloresi” [tiab] OR “G.nipponicum” [tiab] OR “G.malaysiae” [tiab] OR “G.binucleatum” [tiab])

NOT (“Animals” [Mesh] NOT “Humans” [Mesh])

The search term will be adapted for other bibliographic database usage.

The search will be restricted to papers in English, German, French, Spanish or Italian. There will be no geographical restriction nor date limitation.

The searches will be re-run just before the final analyses and further studies retrieved for inclusion.

## Condition being studied

Gnathostomiasis is a food-borne zoonosis, mainly transmitted via the consumption of raw or undercooked fish and meat. The main symptoms consist of migratory cutaneous swellings and eosinophilia.

## Population

Human gnathostomiasis patients

## Intervention

Not applicable

## Comparison/Control

Not applicable

## Types of study to be included initially

No restrictions are set on the types of studies eligible for inclusion. Given the rather broad search approach and the expected limited number of high-quality studies, this review will not only include randomized controlled trials (RCTs) but also consider all other types of published study designs. A thorough quality assessment will be necessary before the final inclusion of the studies. When in doubt, two independent reviewers will be consulted for additional evaluation. The definite final study selection will be determined after completion of the search and quality assessment.

## Primary outcome

Epidemiological, clinical and diagnostic findings in patients with gnathostomiasis, along with data on therapy strategies and clinical outcome are the outcomes of interest in this review. Those are further specified in the data extraction list.

## Secondary outcome

Not applicable

## Measures of effect

A descriptive summary of the frequency of symptoms, laboratory abnormalities, complications or sequelae, and outcomes in human gnathostomiasis will be created. Included variables will be percentages, medians, and ranges. The geographic data collected will be represented as maps.

## Selection strategy

An automatic and manual de-duplication will be performed for the identified material. For the automatic de-duplication the Endnote software and the online tool Covidence will be used. Titles and abstracts of eligible studies identified via searches will be screened and reviewed manually. Furthermore, the reference lists of relevant studies will be manually searched and again screened for matching studies or articles, of which the reference list will also be screened for additional studies. Potential eligible studies will be obtained as full text papers. Studies that do not fulfil the inclusion criteria will be excluded and the respective bibliographic details will be listed in an appendix. Results will be reported using a PRISMA diagram. For storage and processing the bibliographic software ENDNOTE will be used.

## Data extraction

Data from the included studies will be extracted using a standardized, pre-piloted form to evaluate study quality and synthesize evidence. The extracted information will cover study quality, patient characteristics, epidemiological, clinical and laboratory findings, diagnostic methods, treatment regimens, and outcome data. A detailed list of the extracted parameters is provided below:

Study characteristics:

- Reference No. according to our search list
- Year
- First author
- Title
- Journal
- Country of study
- Type of study
- Study Period
- Inclusion / exclusion of article; If exclusion, reason for exclusion
- Number of Human gnathostomiasis cases reported in the reference
- Number of cases already reported
- Cohort study: Human gnathostomiasis out of how many cases of what
- Patient specifity of data (data specified for each patient/Data from cumulation (e.g case series))

Epidemiology

- Clinical Patient's age (years)
- Patient's sex
- Country of acquisition (most likely)
- If imported: Time between end of trip and symptoms
- Country of diagnosis
- Autochthonous or imported case
- Occupational risk factors
- Dietary Risk factors
  - Raw fish/amphibia/birds/Contaminated drinking water
- Year of Acquisition
- Pre-existing medical conditions
- Immunocompromised yes/ no
- Pregnancy
  - Week of pregnancy

Presentation

- Symptomatic/asymptomatic
- Time from symptoms onset to presentation in a hospital/at a physician
- Hospital admission
- Duration of hospitalisation
- General symptoms
- Cutaneous symptoms
  - Location/Body part
- Gastrointestinal symptoms
- Genitourinary symptoms
- Neurological symptoms
- Ocular symptoms
- Other Symptoms

Diagnostics

- Haemoglobin
- Haematocrit
- CRP elevated
  - CRP exact Value
- ESR elevated
  - ESR exact value
- Leukocytosis
  - Leukocytes exact value
- Eosinophilia
  - Eosinophils exact value
- Elevated liver enzymes (at least one)
- AST elevated
  - AST exact Value
- ALT elevated
  - ALT exact Value
- gGT elevated
  - gGT exact Value
- AP elevated
  - AP exact value
- Bilirubin elevated
  - Bilirubin exact value
- Serology
- PCR
- Biopsy
- Gnathostoma species
- CSF
- Imaging (Rx/MRT/CT)
  - Findings
- Coinfections
- Initial diagnosis
- Other laboratory

Treatment

- Larva removal
- Received anthelminthics
- Compound
  - Dosage/Duration
- Corticosteroids
  - Dosage/Duration
- Side effects of treatment
- Other treatment

Outcome

- Complications
- Specify complications
- Outcome
- Specify cause of death
- Sequelae

## Risk of bias (quality) assessment

The potentional eligible full text papers will be assessed for methodological validity prior to inclusion into the review. If doubt occurs, the paper will be analysed by a second reviewer and then discussed. If a disagreement occurs, a third independent reviewer will be included to resolve the problem. As there is a low expected number of high-quality study designs, the focus of this review is set to questions about completeness of data and selective reporting, as the great majority of studies will not be randomised or blinded.

It has to be taken into consideration how the quality assessment results might have an influence on the conclusions and recommendations of the review. This will be presented in the “Discussion” section of the review.

The review itself will be checked using a systematic review quality assessment tool, the PRISMA checklist.

## Strategy for data synthesis

The data will be visually presented in summary tables and synthesized narratively in a sense of an observational analysis. As far as the available data allows, associations and conclusions will be drawn. Where we can, we will attempt to group similar data.

At this stage, the decision will be made whether the data will only be synthesized narratively or if the data is sufficient for a meta-analysis. Four aspects will be assessed whether it is appropriate to combine the results in a meta-analysis.

1: Studies should be similar in terms of the patients (inclusion criteria, patient characteristics)

2: Interventions/Exposures and Comparators should be the same

3: The same outcomes should be reported (primary or secondary, as well as time frames)

4: The results should show that the effects/impacts are generally going into the same direction (visualized by forest plot using a statistical software)

If all four criteria are sufficiently fulfilled by the data from reviewed studies, a meta-analysis will be performed – due to rather expectable lack of sufficient homogeneous data, further planning in this direction is not appropriate now. In case of only some studies meeting all the criteria, it may be considered to perform a meta-analysis only using those studies. In this case, a sensitivity analysis will be carried out, using the remaining studies to test the robustness of the results. Any decisions will be justified in the text of the review, clearly setting out the reasons of why a meta-analysis was performed or not.

As the scoping search showed a relatively limited number of published studies, we may expect limitations due to a lack of data. It is likely in this review that we will have to deal with a variety of different study designs with different study aims, resulting in a rather large heterogeneity. Furthermore, the review question is not just comparing two different interventions on the outcome, we want to get a further picture of human gnathostomiasis. Examining the epidemiology, the clinical impact and laboratory findings, evaluating different therapy plans and outcomes, we will most likely have to deal with a large variety of data or possibly a lack of data. Since one of the aims of our review is to show the current state of knowledge, discovering possible knowledge gaps, this will be considered and discussed in the “discussion” and “conclusions” section.

## Analysis of subgroups or subsets

If the necessary data are available, subgroup analyses will be done for cases from different world regions, age groups, gnathostoma species as well as possible risk factors for poor outcome. As the scope and quality of data is still unclear at this point it is not possible to specify the groups in advance.

## Additional Information

This systematic review will be conducted in line and with the same intention as the below listed systematic reviews previously conducted by our team:

<https://pubmed.ncbi.nlm.nih.gov/39093857/>

https://pubmed.ncbi.nlm.nih.gov/39102427/

https://PubMed.ncbi.nlm.nih.gov/35171908/

https://PubMed.ncbi.nlm.nih.gov/33705384/

https://PubMed.ncbi.nlm.nih.gov/33705387/
