# Supplementary material for: Human gnathostomiasis – A systematic review and analysis of the literature
Source: PLoS Negl Trop Dis. 2026 Jul 31;20(7):e0014546. doi: 10.1371/journal.pntd.0014546 (PMC13426996; doi:10.1371/journal.pntd.0014546)
Supplement: S1 Table — (DOCX) [file pntd.0014546.s005.docx]

Data extraction sheet

| **Year** |
| --- |
| **First author** |
| **Title** |
| **Journal** |
| **Country of study** |
| **Type of study** |
| **Study period** |
| **If excluded but still useful to discussion: specify** |
| **Inclusion / exclusion of article step 2 (full text)** |
| **If exclusion, reason for exclusion** |
| **Specify language** |
| **Number of human gnathostomiasis cases reported in the reference** |
| **Number of cases already imported** |
| **Cohort study: (gnathostomiasis out of how many cases of what)** |
| **Comment** |
| **Patient specificity of data** |
| **No data on epidemiology** |
| **Patient's age (years), if not specific: median** |
| **Patient's sex** |
| **Most likely country of acquisition** |
| **If imported: time between end of trip and symptoms** |
| **Country of diagnosis** |
| **Autochthonous or imported case** |
| **Occupational risk factors** |
| **Dietary risk factor** |
| **Pre-existing conditions** |
| **Immunocompromised** |
| **If immunocompromised specify** |
| **Pregnancy** |
| **If pregnancy: which week** |
| **No data on symptoms** |
| **Symptomatic / asymptomatic** |
| **Data on individual symptoms available** |
| **Time from symptoms to presentation** |
| **Hospital admission** |
| **Duration of hospitalisation** |
| **Number of lesions/larvae** |
| **General symptoms** |
| **CLM (cutaneous larva migrans syndrome)** |
| **Specify CLM** |
| **Location/body part** |
| **VLM (visceral larva migrans)** |
| **Specify VLM** |
| **Organinfiltration** |
| **Location of larva removal** |
| **Git symptoms** |
| **NLM (neurological larva migrans)** |
| **Specify NLM** |
| **Neurological symptoms** |
| **OLM (ocular larva migrans)** |
| **Specify ocular symptoms** |
| **Ocular worm location** |
| **Other symptoms** |
| **No data on diagnostics** |
| **Data on individual laboratory available** |
| **Haemoglobin** |
| **Haematocrit** |
| **CRP elevated** |
| **CRP exact value** |
| **ESR elevated** |
| **ESR exact value** |
| **Leukocytosis** |
| **Leukocytes exact value** |
| **Eosinophilia** |
| **Eosinophils exact value** |
| **Eosinophils percentage** |
| **Elevated liver enzymes (at least one)** |
| **If yes, specify** |
| **Serology** |
| **Serology** |
| **Crude antigen** |
| **Location of laboratory** |
| **PCR from biopsy** |
| **Diagnostic biopsy** |
| **Histological description** |
| **Gnathostoma species** |
| **CSF findings** |
| **Imaging (RX/MRT/CT)** |
| **Imaging findings** |
| **Initial diagnosis** |
| **Other diagnostics** |
| **No data on treatment** |
| **Larva removal (fully or partial)** |
| **Treatment induced eruption of parasite** |
| **Received anthelminthics** |
| **Compound** |
| **Daily dosage** |
| **Duration** |
| **Systemic corticosteroids** |
| **Compound** |
| **Dosage** |
| **Duration** |
| **Retreatment** |
| **Retreatment 1 compound** |
| **Daily dosis** |
| **Duration** |
| **Retreatment 2 compound** |
| **Daily dosis** |
| **Duration** |
| **Side effects of treatment** |
| **Other treatment** |
| **Relapse** |
| **Nr. Of relapses** |
| **No data on outcome** |
| **Complications** |
| **Specify complications** |
| **Outcome** |
| **Specify cause of death** |
| **Sequelae** |
| **Comment** |
| **Syndrome shift** |

CLM location code template:


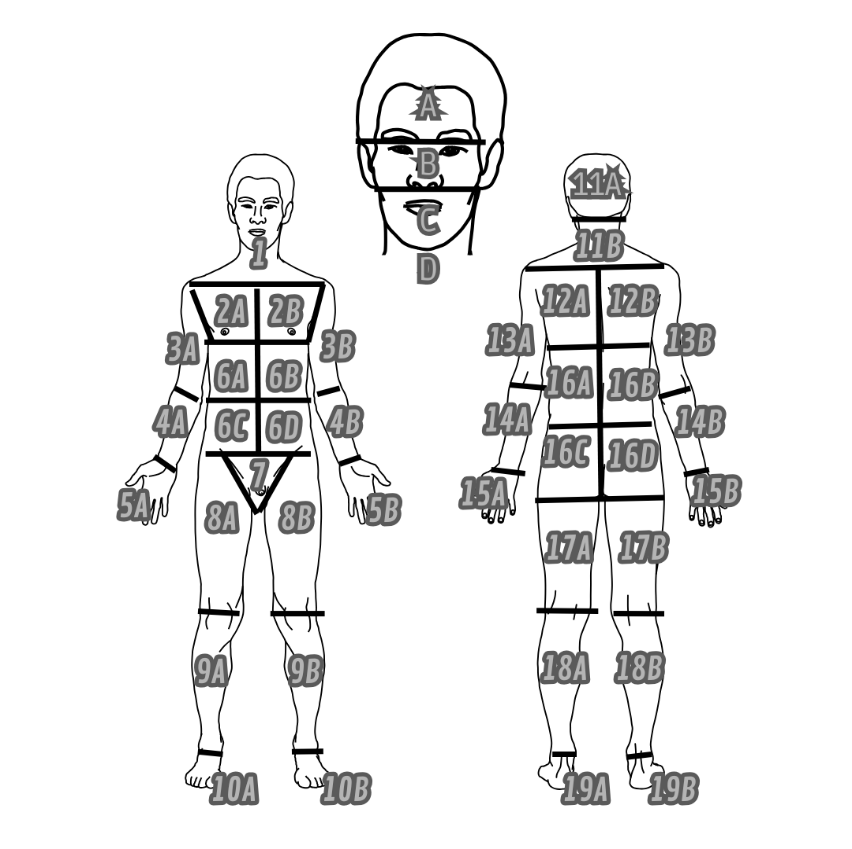


[Body template adapted from an open source clipart (Link to source: https://openclipart.org/detail/314881/human-male-and-female-body-line-art)].
