# Supplementary material for: Human gnathostomiasis – A systematic review and analysis of the literature
Source: PLoS Negl Trop Dis. 2026 Jul 31;20(7):e0014546. doi: 10.1371/journal.pntd.0014546 (PMC13426996; doi:10.1371/journal.pntd.0014546)
Supplement: S1 Fig — (PDF) [file pntd.0014546.s008.pdf]

Western blot based on crude antigen preparation of *G. spinigerum* advanced 3rd stage larvae

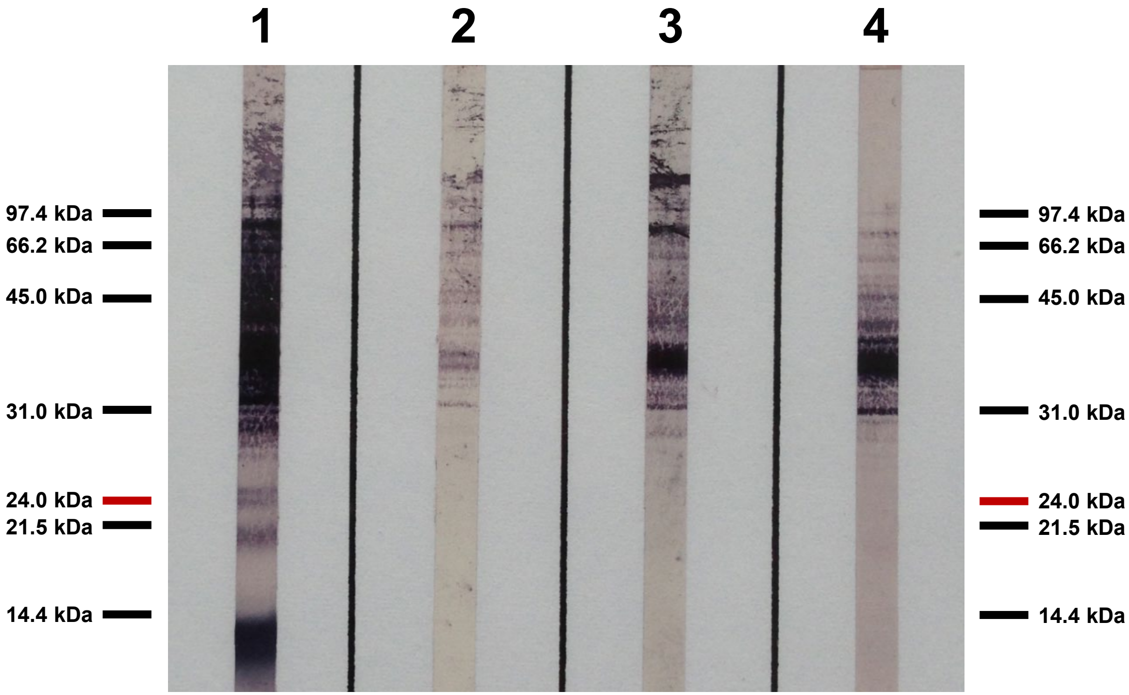

1 = Positive control Lao PDR:	positive
2 = Negative control:	negative
3 = North American patient:	negative
4 = Positive control Peru:	negative

Neumayr A, Ollague J, Bravo F, Gotuzzo E, Jimenez P, Norton S, et al. Cross-reactivity pattern of Asian and American human gnathostomiasis in Western Blot assays using crude antigens prepared from *Gnathostoma spinigerum* and *G. binucleatum* third-stage larvae. Am J Trop Med Hyg. 2016;95(2):413-6.

Western blot based on crude antigen preparation of *G. binucleatum* advanced 3rd stage larvae

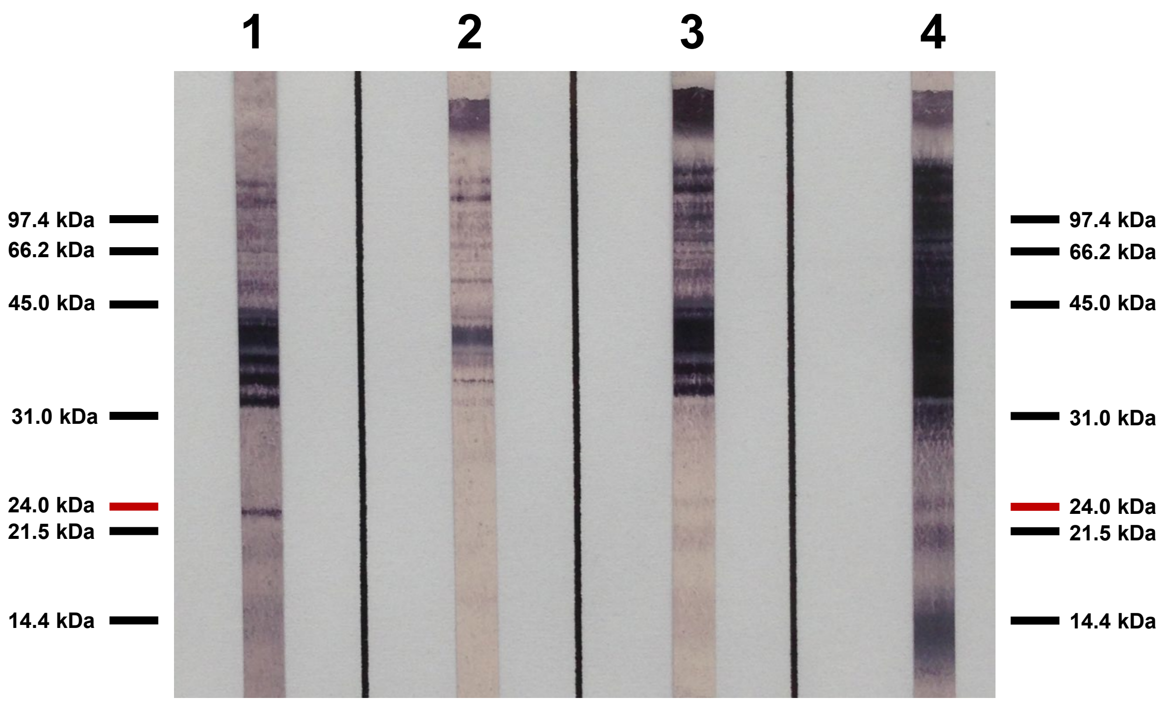

1 = Positive control Peru:	positive
2 = Negative control:	negative
3 = North American patient:	positive
4 = Positive control Lao PDR:	positive

Neumayr A, Ollague J, Bravo F, Gotuzzo E, Jimenez P, Norton S, et al. Cross-reactivity pattern of Asian and American human gnathostomiasis in Western Blot assays using crude antigens prepared from *Gnathostoma spinigerum* and *G. binucleatum* third-stage larvae. Am J Trop Med Hyg. 2016;95(2):413-6.
